# Transcriptomic analysis implicates ABA signaling and carbon supply in the differential outgrowth of petunia axillary buds

**DOI:** 10.1186/s12870-023-04505-3

**Published:** 2023-10-10

**Authors:** Zhiwei Luo, Dan Jones, Sarah Philp-Wright, Joanna Putterill, Kimberley Cathryn Snowden

**Affiliations:** 1 The New Zealand Institute for Plant and Food Research Limited, Auckland, New Zealand; 2 School of Biological Sciences, University of Auckland, Auckland, New Zealand; 3 NetValue Limited, Hamilton, New Zealand

**Keywords:** Axillary buds, Branching, Abscisic acid, Strigolactones, Phosphate, Bud dormancy

## Abstract

**Background:**

Shoot branching of flowering plants exhibits phenotypic plasticity and variability. This plasticity is determined by the activity of axillary meristems, which in turn is influenced by endogenous and exogenous cues such as nutrients and light. In many species, not all buds on the main shoot develop into branches despite favorable growing conditions. In petunia, basal axillary buds (buds 1–3) typically do not grow out to form branches, while more apical axillary buds (buds 6 and 7) are competent to grow.

**Results:**

The genetic regulation of buds was explored using transcriptome analyses of petunia axillary buds at different positions on the main stem. To suppress or promote bud outgrowth, we grew the plants in media with differing phosphate (P) levels. Using RNA-seq, we found many (> 5000) differentially expressed genes between bud 6 or 7, and bud 2. In addition, more genes were differentially expressed when we transferred the plants from low P to high P medium, compared with shifting from high P to low P medium. Buds 6 and 7 had increased transcript abundance of cytokinin and auxin-related genes, whereas the basal non-growing buds (bud 2 and to a lesser extent bud 3) had higher expression of strigolactone, abscisic acid, and dormancy-related genes, suggesting the outgrowth of these basal buds was actively suppressed. Consistent with this, the expression of ABA associated genes decreased significantly in apical buds after stimulating growth by switching the medium from low P to high P. Furthermore, comparisons between our data and transcriptome data from other species suggest that the suppression of outgrowth of bud 2 was correlated with a limited supply of carbon to these axillary buds. Candidate genes that might repress bud outgrowth were identified by co-expression analysis.

**Conclusions:**

Plants need to balance growth of axillary buds into branches to fit with available resources while allowing some buds to remain dormant to grow after the loss of plant parts or in response to a change in environmental conditions. Here we demonstrate that different buds on the same plant with different developmental potentials have quite different transcriptome profiles.

**Supplementary Information:**

The online version contains supplementary material available at 10.1186/s12870-023-04505-3.

## Background

Shoot branching is a key determinant of the shape of a plant and is a dynamic, plastic and tightly regulated process. Plants modulate this process to achieve optimal growth and sometimes to survive. Although more branches could increase the amount of energy harvested and potentially improve yield, branching is a costly process that must consider nutrient availability, environmental conditions, and information from throughout the plant, such as the presence or absence of other growing shoots [1, 2].

Axillary meristems develop on the adaxial side of the leaf axils, and their initiation involves a complex network that involves hormone signalling, transcriptional regulation, protein movements and interactions, and feedback regulation in multiple pathways [3–5]. Once these axillary meristems develop, they can grow out with little delay or become dormant. Dormant buds can become active at later stages of development or stay dormant depending on environmental cues, such as temperature, day length, and nutrient levels. In many species, the growth of axillary meristems on the main stem is different depending on their position. For instance, in garden petunia (*Petunia hybrida*), the axillary meristems at the axil of the cotyledons and the first two leaves do not usually grow to form branches, while the meristems from the more apical nodes grow soon after the leaf at that node is fully expanded [6, 7]. Thus, the outgrowth of axillary meristems is the consequence of a series of interconnected, and often competing, signals (and the pathways they trigger) from both outside and inside the plant.

Phytohormones, including auxin, abscisic acid (ABA), cytokinin (CK), strigolactones (SLs), as well as sugars play a role in regulating axillary meristem outgrowth [4, 5, 8–13]. SLs are a group of branching inhibitory hormones that serve as a core component of signalling and regulatory networks of branching. For example, SL biosynthetic mutants with lower SL levels display increased branching in several species [7, 14–16]. The core SL biosynthesis and signalling pathways are conserved in many species including model systems and woody perennial plants [2, 5]. The inhibitory effect of SL on branching is mediated through direct binding of the hormone to the receptor DECREASED APICAL DOMINANCE2 (DAD2)/DWARF14 (D14)/RAMOSU3 (RMS3), and its F-box interaction partner, MORE AXILLARY GROWTH2 (MAX2)/RMS4/D3. This results in the degradation of transcriptional repressor D53/ SUPPRESSOR OF MORE AXILLARY GROWTH1-LIKEs (SMXL6,7,8), which in turn increases the transcription of genes encoding transcription factors (TFs), such as BRANCHED1 (BRC1) and IDEAL PLANT ARCHITECTURE1 (IPA1) [17–20]. Recent studies suggested that SMXLs also function as TFs that suppress their own transcription [21, 22].

*BRC1* in Arabidopsis and its homologs from other species are expressed in axillary buds and encode branching repressors. Their expression is regulated by multiple mechanisms, including SL and CK signalling, decapitation, sucrose treatment, nutrient availability, and light quality. Thus, *BRC1* is thought to be an integrator for various branching signals [4, 5, 23]. However, there is also evidence for *BRC1* and/or SL independent regulation of branching. For instance, the Arabidopsis *brc1brc2* double mutant grown in low nitrogen (N) media or in media containing 5 µM GR24, a synthetic SL analogue, had fewer total branches compared with those grown in high N media or in media without GR24 [24]. Similarly, the SL biosynthesis mutant *carotenoid cleavage dioxygenase8* (*ccd8*) and the *dad2* mutant in petunia also had fewer branches in low P than high P conditions [25]. The BRC1/SL independent pathway(s) are yet to be fully identified and characterised; however, they likely involve other hormones. Auxin and CK have long been suggested as important players in shoot branching regulation [4, 8]. In recent years, multiple studies have suggested ABA also regulates shoot branching, especially under a low red:far red (R:FR) light ratio [10, 26, 27].

The growth of axillary buds is very dynamic in response to environmental signals. Previous studies from our lab have shown that modulating nutrient levels and the R:FR ratios of light lead to a range of shoot branching outcomes in petunia, from a high degree of axillary meristem activity to strong suppression of branching [25]. Nutrient and light quality regulation of branching have been studied by many researchers [5, 28–30]. Low P increased SL levels and reduced branching [12, 25, 31].

Here, we used *Petunia hybrida*, a perennial species from the Solanaceae family to study the transcriptome differences between the axillary buds that typically have different growth outcomes on the main stem. In petunia, the basal axillary buds (buds 1 and 2, and to a lesser extent bud 3) on the main stem rarely produce branches, while the more apical axillary buds (e.g., buds 6 and 7) almost certainly will grow out in growth-promoting conditions. One interesting aspect about these basal axillary buds is that they tend to grow out and form a branch in SL mutants or after decapitation ([7] and Figure S1A). We hypothesized that the regulation of outgrowth between axillary buds at different positions differs and there are additional mechanisms that regulate branching under different nutrient conditions. We have identified many differentially expressed genes (DEGs) between bud 2 and bud 6, which coincided with our phenotypic observations of bud outgrowth, and found the transcript levels of ABA associated genes were affected by P level within 24 h of switching from low P to high P conditions, implying ABA might contribute to branching suppression in a low P environment. In addition, comparison between this transcriptome data and data from other species suggested that growth suppression of basal axillary buds was correlated with the limited supply of carbon to these axillary buds.

## Results

### The outgrowth of axillary buds differs depending on their position, and phosphate supply alters the outgrowth of the apical, but not basal axillary buds in petunia

**Fig. 1:**
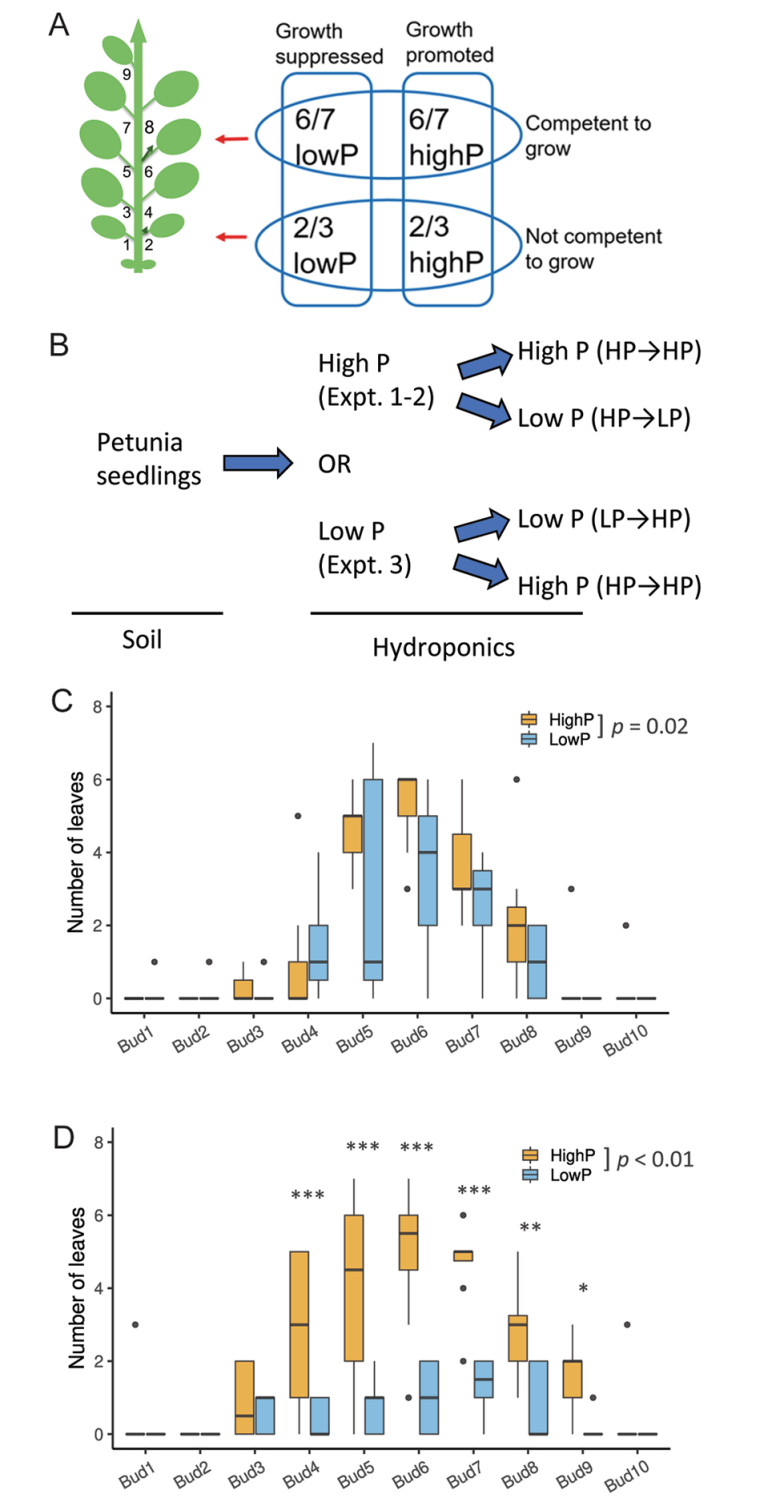
Growth of axillary buds is dependent on their position and nutrient level. **A**, experimental system to compare basal (e.g., buds 2–3) and apical axillary buds (e.g., buds 6–7) under growth promoting (high phosphate, high P) or growth suppressing (low P) conditions based on our previous findings [25]; **B**, experimental design for the three experiments that were carried out for axillary bud sample collection; **C** and **D**, apical axillary buds had more growth compared with basal axillary buds. C, branch growth in experiment two; D, branch growth in experiment three. Low P conditions suppress (C) while high P conditions promote (D) the growth of apical axillary buds. The numbers of leaves at each bud were counted 7 days after medium changes. The bottom and top sides of the box represent the first and third quantiles and the line inside the box represents the median of the data, and the outliners are the dots above the whiskers (n = 7–8). A generalized linear model was fitted with the data and the statistical significance of the overall P treatment and P effect on each bud was calculated using Analysis of Variance (ANOVA) and Turkey’s honestly significant difference (HSD), and the level of significance is indicated as follows: *, *p* < 0.05; **, *p* < 0.01; and ***, *p* < 0.001

Our previous work showed that the growth of petunia axillary buds can be altered by different combinations of light quality (R:FR) and phosphate (P) levels [25] (summarized in Fig. 1A). To examine the transcriptional differences between buds along the main shoot, experiments were designed that considered bud position and therefore developmental potential, and the presence of a stimulus either to grow or to suppress growth. We compared buds that are responsive to environmental changes affecting growth (buds 6 and 7, referred to here as apical axillary buds), with buds that do not respond to environmental conditions (buds 2 and 3, referred to here as basal axillary buds). We decided to use changes in P levels in the growing medium to stimulate or suppress growth of the axillary buds (Figs. 1A and B). Interestingly, buds 2 and 3 can grow out and produce a branch after decapitation (Figure S1A), suggesting environmental conditions may not be the dominant factor for control of branching from basal axillary buds.

In the first two independent experiments, soil germinated and grown petunia seedlings were placed in a complete hydroponic solution including 250μM P (referred to as high P or HP hereafter), mimicking standard growing conditions, before being split into two groups. One group of plants was transferred into fresh high P medium, whilst the other group was put into a hydroponic solution with reduced levels of P (5 µM P, referred to as low P or LP) (Fig. 1B). The branch growth at each node was measured 7 days after transferring into new conditions. The branching phenotypes resulting from these two experiments were very similar (Figure S1B, Fig. 1C, and Figure S1C). As expected, the apical axillary buds (e.g., buds 6 and 7) had more branch growth when compared with the basal axillary buds (e.g., bud 2 and bud 3) (Fig. 1C and Figure S1B). Plants that were transferred to a low P condition had reduced branch growth overall, mainly on the apical axillary buds, compared with plants that remained in high P medium (Fig. 1C). Although the differences in branch growth from each bud between the two treatments were not statistically significant, the P effect on overall branch growth was significant (*p* = 0.02) (Fig. 1C).

In the third experiment, soil germinated seedlings were placed in low P medium before being divided into two groups. One group went into fresh low P medium whilst the other group was transferred into high P medium (Fig. 1B). Under low P conditions, buds 6 and 7 had more growth than bud 2 and bud 3 (Fig. 1D). However, the difference was smaller than the growth difference between buds 6 and 7 and buds 2 and 3 in the first and second experiments where plants started with high P conditions (Fig. 1C and Figure S1B). We found a more profound effect of shifting to higher P on the growth of axillary buds compared with the low P effect on suppressing bud growth in the first and second experiments, and again the effect was mainly observed at the apical axillary buds (Fig. 1D). In addition to the significant (*p* < 0.01) effect of high P on overall branch growth compared with low P conditions, the disparity in branch growth between treatments was also significant for several bud positions (from bud 4 to bud 9) (Fig. 1D and Figure S1C). Interestingly, in the third experiment bud 3 had some growth (Fig. 1D), which was not observed in the first and second experiments (Fig. 1C and Figure S1B).

### Apical and basal axillary buds have distinct transcriptome profiles

The different growth outcomes for axillary buds along the main stem suggest the branching regulation for an individual bud may be different. To examine the differences between these apical and basal axillary buds at the transcriptome level, we conducted RNA-seq experiments using axillary bud samples. As our focus was primarily on bud position and developmental difference, and not P responses, early time points (3 and 24 h) were chosen before large changes in P status could occur in the plants.

The petunia axillary buds from nodes 2, 3, 6 and 7 were collected at 3 and 24 h after changing the medium in all three hydroponic experiments. A portion of the cDNA from bud 3 and bud 6 samples at the 24 h time-point were analyzed by digital droplet PCR to determine whether the positional difference (bud 3 vs. bud 6) and the effect of switching P medium (high P to low P in the first experiment, and low P to high P in the third experiment) could be detected. The transcript levels of petunia *TCP3* (*Teosinte branched1 [TB1], CYCLOIDEA, and PROLIFERATING CELL FACTOR3*), *DAD2*, *PhPT1* (*PHOSPHATE TRANSPORTER1*), and *CDKB1* (*CYCLIN DEPENDENT KINASE B1*) were measured. *DAD2* encodes the SL receptor [19] and *PhTCP3* is likely a homolog of Arabidopsis *BRC1* [25]. These genes are key regulators in SL signaling and inhibition of shoot branching. *PhPT1* is a P transporter originally found to be expressed in above-ground tissues and is upregulated when P content decreases in petunia petals [32]. *CDKB1* is a cell cycle progression marker and is highly expressed in growing tissues [33]. We found that there were clear differences in the transcript levels of these genes between bud 3 and bud 6, especially in the first experiment (Figure S2). The transcription of *DAD2*, *TCP3* and *CDKB1* (Figure S2A) were consistent with our previous report, where the transcript abundances of *DAD2* and *TCP3* were increased in bud 2 relative to bud 7, while the transcripts of *CDKB1* were more abundant in bud 7 [25].

Twenty-four hours of low P treatment had a minimal effect on expression of the genes mentioned above compared with the plants that stayed in the high P condition in the first experiment (Figure S2A). However, a high P effect was seen in the third experiment, where the plants were placed initially in the low P medium and then shifted to high P (Figure S2B). The transcript levels of two P starvation-responsive genes, *SPX DOMAIN GENE2* (*SPX2*) and *SPX3*, were also quantified: the expression of these genes was 4- to 34-fold higher in the low P condition compared with 24 h of high P (Figure S2C).

**Fig. 2:**
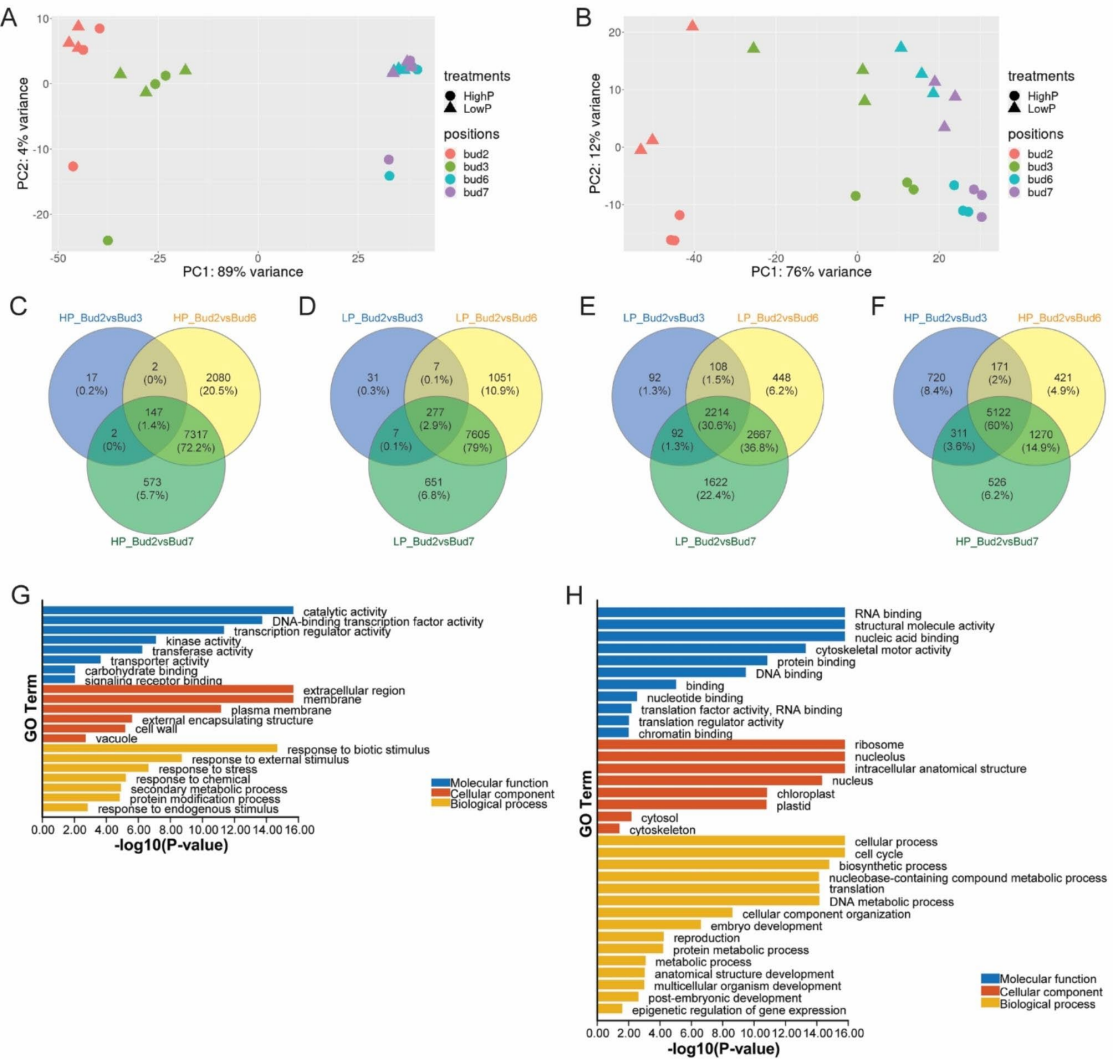
RNA-seq analysis of axillary buds at different positions support their phenotypic differences. **A** and **B**, Principal component analysis (PCA) plots from the 24 h time point of the first experiment (A, plants were started in high phosphate (High P)), and the third experiment (B, plants were started in low phosphate (Low P)). **C-F**, Venn diagrams showing the overlap of significant differentially expressed genes (DEGs) between bud 2 and other buds 24 h after medium changes. In the first experiment, plants were initially grown in high P then either transferred to fresh high P (HP) medium (C) or into fresh low P (LP) medium (D). In the third experiment, plants were initially grown in low P then either kept in LP (E) or transferred into HP medium (F). **G-H**, Gene ontology (GO) enrichment analysis from the DEGs between bud 2 and bud 6 under high P in the first experiment. Significant GO terms for the genes whose transcripts were more abundant in bud 2 compared with bud 6 (G) and more abundant in bud 6 compared with bud 2 (H)

RNA-seq was carried out on samples from the first (Figure S1B) and the third experiment (Fig. 1D). When analyzing the 24 h time-point samples, principal component analysis (PCA) of all the transcripts showed that buds 6 and 7 were clustered together closely whilst bud 2 was different from other buds (Fig. 2A and B). The PCA plots also implied bud 3 was intermediate between bud 2 and buds 6 and 7, clustering closer to bud 2 in the first experiment (high P shifted to low P) (Fig. 2A) but closer to buds 6 and 7 in the third experiment (low P shifted to high P) (Fig. 2B). This was consistent with the phenotypic data where there was a small amount of growth from bud 3 in the third experiment (Fig. 1D) compared with bud 3 in the first and second experiments (Figure S1B and Fig. 1C). In terms of a P effect, the PCA analysis could not distinguish the low P treatment effect 24 h after the medium was switched in the first experiment (Fig. 2A), whereas the high P effect in the third experiment was distinct (Fig. 2B).

The DEGs (fold changes > |2| with padj < 0.05) analysis also indicated a substantial difference between bud 2 and other buds, especially buds 6 and 7 (Fig. 2C-F and Table S1). In the first experiment, there were more than 8000 significant DEGs between bud 2 and bud 6 or 7 from plants that either remained in high P or were transferred to low P medium for 24 h (Fig. 2C and D, Table S1). We did not see much difference in the number of DEGs between the two P conditions in buds 2, 3, 6 and 7, which may indicate that plants retain P reserves after 24 h of low P. Transcripts that were upregulated in bud 6 or 7 relative to bud 2 included many growth- and cell cycle-related genes; whereas the genes that had increased transcripts in bud 2 compared with bud 6 or 7 included a large number of stress, defense, and hormone related genes and gene families. For example, genes from the *LATE EMBRYOGENESIS ABUNDANT* (*LEA*) family, *NAM*, *ATAF1/2*, *CUC2* (*NAC*) family, *WRKY* family genes, ABA and ethylene associated genes were upregulated in bud 2 (Table S1). Focusing on the genes common to all three sets of DEGs (bud 2 vs. buds 3,6,7) from the first experiment (Fig. 2C and D), there were several genes associated with auxin, ABA, and CK response and catabolism (Table S2). For example, *PIN-FORMED6* (*PIN6*) was upregulated in buds 3, 6, 7 relative to bud 2, while the transcript abundances of *CYTOKININ DEHYDROGENASE3/5* (*CKX3* and *CKX5*) were elevated in bud 2 compared with other buds. Some of these genes such as *CKX3/CKX5*, *PROTODERMAL FACTOR1* (*PDF1*), and *TORNADO2* (*TRN2*) have been suggested to be involved in shoot meristem development in other studies [34–36].

In the third experiment (low P to high P), the number of significant DEGs between bud 2 and bud 6 or 7 when the plants were started in low P and stayed in low P was large (~ 5000, Fig. 2E), but lower in number than in the first experiment (Fig. 2C, > 8000 DEGs). However, the number of DEGs between bud 2 and bud 6 or 7 increased to around 7000 for the plants that were transferred from low P to high P medium for 24 h (Fig. 2F). Although many of the growth- and cell cycle-related transcripts were still more abundant in buds 6 and 7 in low P, the magnitude of the difference was reduced compared with the same set of genes under high P conditions in the first experiment. For example, the transcripts of *CDKB1* in buds 6 and 7 were approximately 4-fold more abundant than in bud 2 exposed to high P conditions in the first experiment; but were only about 2-fold more abundant under low P conditions in the third experiment compared with the levels in bud 2 (Figure S2D). These results suggested that low P conditions might suppress the expression of many genes that would otherwise be differentially expressed between bud 2 and bud 6 or 7, and it appeared the plants were more responsive to high P after a prolonged period of low P conditions. These results could explain the growth difference observed between apical axillary buds in the high to low P (first and second) and low to high P (third) experiments. The significant DEGs observed in buds 2, 3, 6, and 7 from plants in low P compared with high P in the third experiment indicated that switching to high P had a large effect on axillary buds, especially buds 3, 6 and7, whereas bud 2 was less responsive to high P after 24 h (Figure S3A, Table S3). There are 111 common genes among these four sets of DEGs including several P starvation genes and stress-associated genes, most of these (108) common genes were downregulated by 24 h of high P condition and only three (*LOB DOMAIN-CONTAINING PROTEIN38* (*LBD38*), *RNA POLYMERASE III RPC4*, and *ARGININE METHYLTRANSFERASE11* (*PRMT11*)) were upregulated by high P in all buds (Figure S3A and Table S3). Focusing on the common genes between low and high P on bud 3, 6, and 7, there were 210 common genes with most being downregulated by high P treatment, including a few ABA- and senescence-related genes (Table S3).

We also compared the DEGs between the bud 3 samples of these two RNA-seq experiments given the differences in the growth of these buds from the different experiments (Figure S1B and Fig. 1D), and surprisingly found a large number of significant DEGs. There were about 4700 DEGs when comparing the transcripts from bud 3 under high P in the first experiment with bud 3 under low P in the third experiment (the starting conditions for each experiment) (Table S4). The number of DEGs between bud 3 under high P conditions in the first experiment and bud 3 under 24 h of high P in the third experiment (that started with low P and then went into high P) increased to more than 8000 (Table S5). These results showed bud 3 from these two experiments differed substantially.

Gene ontology (GO) enrichment analyses were employed to give an overview of the representation of genes that were highly expressed in bud 2 or bud 6, respectively, in both experiments. The GO enrichments for the 24 h time-point between bud 2 and bud 6 were very different (Fig. 2G and H, and Figures S3B and S3C). Some of the significant GO terms in bud 2 included stress-related processes, and catalytic and transcription activities. The significant GO terms for genes upregulated in bud 6 included cell cycle, RNA/DNA binding, and ribosome. The enrichment terms for bud 2 and bud 6 were similar for both transcriptome experiments (Fig. 2G and S3B for bud 2, and Fig. 2H and S3C for bud 6).

### SL, auxin, CK and sugar related genes were expressed differentially between axillary buds at different positions

**Fig. 3:**
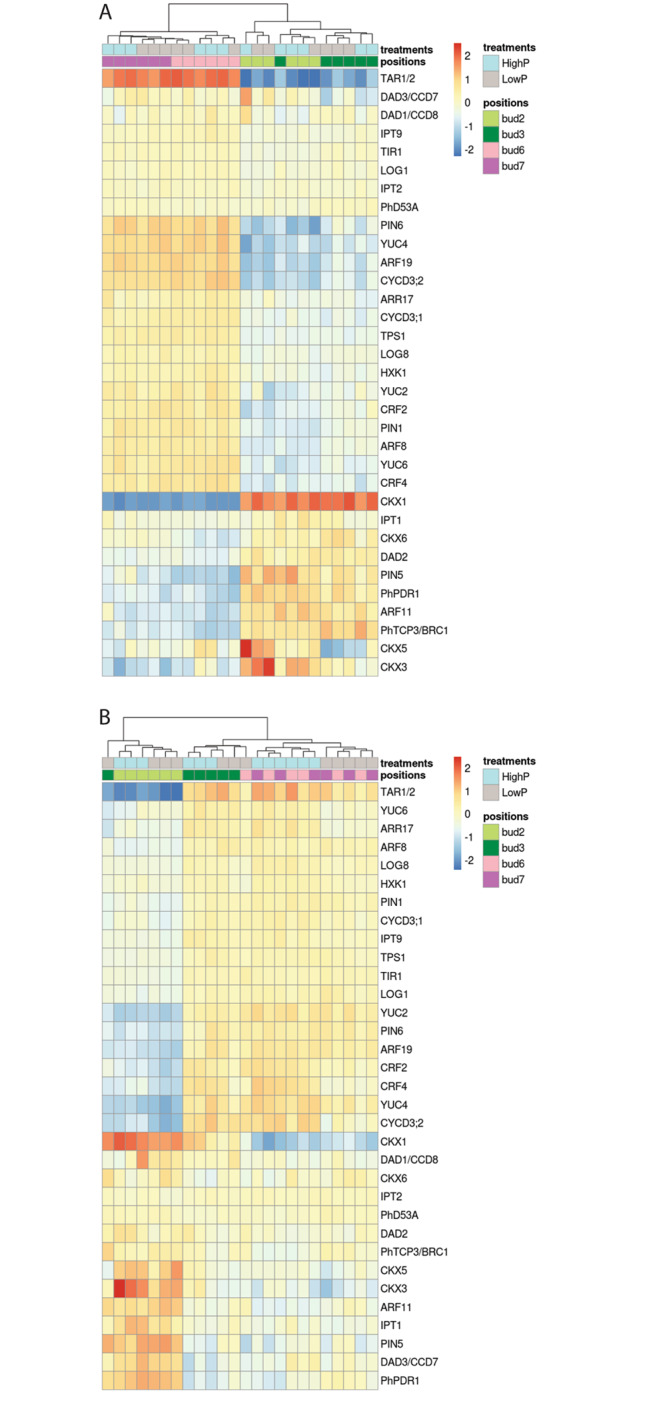
Hormone- and sugar-related genes were expressed differentially by axillary buds at different positions. Selected genes include auxin biosynthesis genes (*YUCCA*s (*YUC2*, *4*, *6*); *TRYPTOPHAN AMINOTRANSFERASE RELATED1*/*2* (*TAR1*/*2*)), auxin efflux carriers (*PIN-FORMED*s (*PIN1*, *5*, *6*)), auxin receptor (*TRANSPORT INHIBITOR RESPONSE1* (*TIR1*)), auxin response genes (*AUXIN RESPONSE FACTOR*s (*ARF8*, *11*, *19*)), cyclins (*CYCD3;1* and *CYCD3;2*), cytokinin (CK) biosynthesis genes (*ISOPENTENYLTRANSFERASE*s (*IPT1*, *2*, *9*); *LONELY GUY*s (*LOG1*, *3*)), CK response genes (*RESPONSE REGULATOR17* (*ARR17*); *CYTOKININ RESPONSE FACTOR*s (*CRF2*, *4*)), CK degradation genes (*CKX1*, *3*, *5*, *6*), SL biosynthesis genes (*CCD7*, *8*), strigolactones (SLs) signaling genes (*DAD2*, *D53A, PhTCP3*/*BRC1*), SL transporter (*PhPDR1*), sugar signaling (*HEXOKINASE1* (*HXK1*)), and Trehalose biosynthesis (*TREHALOSE-6-PHOSPHATE SYNTHASE1* (*TPS1*)). Expression heatmaps from the first experiment (high to low phosphate (P; **A**) and the third experiment (low to high P; **B**). The normalised counts of these genes were transformed using the rlog function in DESeq2 package, and the pheatmap package was used for the heatmap construction

Plant hormones and their interactions contribute to every developmental aspect of a plant, including shoot branching. As the branching pattern and transcriptome profiles were found to be very different between axillary buds, we hypothesized that the hormone-related gene expression should reflect these differences. We used the transcriptome data to investigate the expression of a group of selected genes related to hormone synthesis, signaling, and response between the basal and apical axillary buds (mainly between bud 2 and bud 6). Differences were observed for many of the hormone biosynthesis and/or signaling genes at different bud positions (particularly bud 6 or 7 versus bud 2 or 3 in experiment 1, and for bud 2 versus other buds in experiment 3, see Fig. 3).

SL inhibits shoot branching and therefore its biosynthesis and signaling play a key role in the branching regulation of a plant [2, 5, 12, 13]. The transcripts of SL biosynthetic, transport, and signaling genes were thus investigated: the SL transporter, *PLEIOTROPIC DRUG RESISTANCE1* (*PhPDR1*), the receptor *DAD2* and the transcription factor *PhTCP3* were elevated in bud 2 compared with bud 6 and bud 7 from the first experiment (Fig. 3A). In the third experiment, *PhPDR1* transcript levels were higher in bud 2 compared with buds 6 and 7 (Fig. 3B). Although the biosynthetic genes *CCD7* and *CCD8* are more highly expressed in other tissues [25], some differences in transcript levels were also observed in buds, for example *CCD7* had higher transcript levels in bud 2 than other buds (Fig. 3).

CK has been shown to be a positive regulator of shoot branching [11, 37, 38]. In our data, several changes in transcript abundance were observed that are consistent with reduced CK activity in bud 2 and increased CK activity in buds 6 and 7 (Fig. 3). The differences in expression of CK biosynthesis genes were generally low in magnitude (Fig. 3, *ISOPENTENYLTRANSFERASE (IPT)* and *LONELY GUY (LOG)* genes). However, the *CKX* genes, which degrade CK [39–41], were significantly upregulated in bud 2 compared with bud 6 (Fig. 3). In addition, several CK signaling and response genes, such as *CYTOKININ RESPONSE FACTORs* (*CRF2s* and *CRF4s*), type-A *ARABIDOPSIS RESPONSE REGULATOR* (*ARR17*), and cyclins (*CYCs*) were upregulated in buds 6 and 7 compared with bud 2 in experiments 1 and 3 (Fig. 3). In the third experiment, the transcripts of *LONELY GUY8* (*LOG8*) and several CK induced genes increased in bud 6 and/or bud 7 after 24 h of high P (Figure S4), suggesting that CK signaling was regulated at least partially by nutrient level and the response was relatively rapid.

Auxin from the shoot apex has long been suggested to have an inhibitory effect on bud outgrowth; however, once axillary buds start growing, they also become a source of auxin [11, 42]. We found auxin-associated genes, such as *YUCCAs*, *PIN1* and *PIN6*, all had significantly more transcript counts (> 2-fold) in buds 6 and 7 compared with bud 2 (Fig. 3). However, unlike other *PINs*, *PIN5*, an IAA downregulated gene [43], was highly expressed in bud 2 in both experiments (Fig. 3). These data indicated buds 6 and 7 had more auxin signaling occurring than bud 2, aligned with the suggestion that auxin in axillary buds is associated with bud outgrowth.

Studies in several species have identified a role for sugars in regulation of shoot branching [44–47]. In the data presented here, the transcripts of *TREHALOSE-6-PHOSPHATE SYNTHASE 1* (*TPS1*), a trehalose biosynthesis enzyme, and *HEXOKINASE1* (*HXK1*), a glucose sensor, were higher in buds 6 and 7 compared with bud 2 (> 2-fold) in the first experiment (Fig. 3A), consistent with their proposed function of promoting branching in pea and Arabidopsis [48, 49].

### ABA associated genes were more highly expressed in basal axillary buds and expression is regulated by phosphate level

**Fig. 4:**
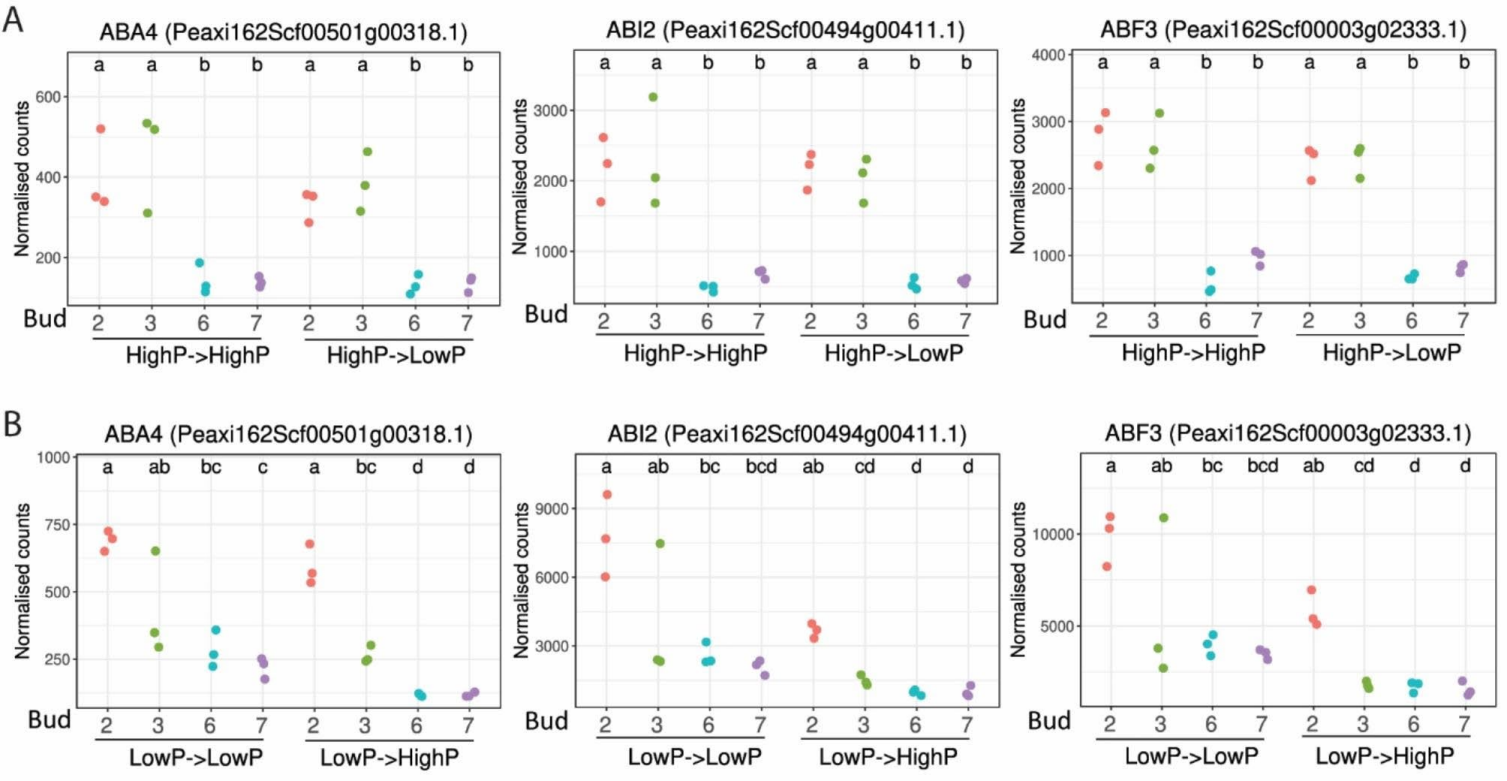
Abscisic acid (ABA)-associated genes were upregulated in basal axillary buds and expression is regulated by phosphate level. **A** (top panel), transcript levels from the first experiment (high to low phosphate), in which the plants started with high P medium then either transferred to new high P medium (HP->HP) or were changed to low P medium (HP->LP). **B** (bottom panel), transcript levels from the third experiment (low to high phosphate), in which the plants started with low P medium then either transferred to new low P medium (LP->LP) or were changed to high P medium (LP->HP). Selected genes include *ABA-DEFICIENT4* (*ABA4*), *ABA INSENSITIVE2* (*ABI2*), and *ABA RESPONSIVE ELEMENTS-BINDING FACTOR3* (*ABF3*). The normalized counts were obtained from the R package DESeq2. The different letters refer to the significance (*p* < 0.05) between samples calculated with Tukey honest significant differences method (Tukey HSD).

ABA is often considered a stress and dormancy hormone, and in the past few years, several reports suggested ABA might regulate shoot branching [9, 10, 27, 50–52]. However, there have been few studies on the relationship between ABA and P level in the plant until recently. Zhang et al. [53] found low P (1 µM) increased the expression of ABA biosynthesis and ABA responsive genes in Arabidopsis seedlings. From our RNA-seq data, we found that not only ABA biosynthesis genes but also the ABA response genes were significantly upregulated in bud 2 compared with buds 6 and 7, suggesting that ABA levels might be lower in apical axillary buds. For example, the *ABA-DEFICIENT 4* (*ABA4*), *ABA INSENSITIVE2* (*ABI2*), and *ABA RESPONSIVE ELEMENTS-BINDING FACTOR3* (*ABF3*) all had significantly more transcripts (> 2-fold) in bud 2 compared with buds 6 and 7 from experiments 1 and 3 (Fig. 4). There were also differences in expression for these genes between low P and high P conditions in the third experiment, especially in buds 6 and/or bud 7 (Fig. 4B). Many other ABA biosynthesis and ABA or stress-induced genes also had significantly elevated (> 2-fold) transcript levels in bud 2 in contrast with bud 6 or bud 7 in at least one of the experiments (Figure S5). In general, the transcripts of these genes were reduced significantly in the apical axillary buds after 24 h of high P compared with the low P treatment in the third experiment (Figure S5B), which is consistent with the findings from Zhang et al. [53]. The responses to changing P level of so many ABA-related genes might explain, to some extent, why the branching pattern of petunia SL mutants were affected by P level [25]. The greater transcript abundance of ABA-related genes in bud 2 and to a lesser extent bud 3 and the rapid (24 h) reduction of these transcripts mainly in buds 6 and 7 after changing from low P to high P, support the role of ABA in branching inhibition and suggest that bud 2 might be in a state of stress and/or dormancy. It is interesting that signaling for multiple hormones (ABA, CK, and SL) showed responses to changing P levels.

### Comparison of the petunia axillary bud DEGs with DEGs identified in dormancy studies in other species

To further examine the growth status of bud 2, comparisons were performed between this petunia data and several published transcriptome datasets that studied dormancy in both model and crop species. In Arabidopsis, 78 genes were identified that were upregulated in dormant buds relative to active buds [47, 54]. We used BLASTp to identify *P. axillaris* homologs of these genes and found that 44 of these genes were differentially expressed and upregulated in bud 2 from at least one of our experiments (Table S6), including the dormancy marker, *DORMANCY-ASSOCIATED PROTEIN-LIKE1* (*DRM1*) (Figure S6) and ABA-associated genes, such as *NINE-CIS-EPOXYCAROTENOID DIOXYGENASE3* (*NCED3*) (Figure S5), and *ABF3* (Fig. 4).

**Fig. 5:**
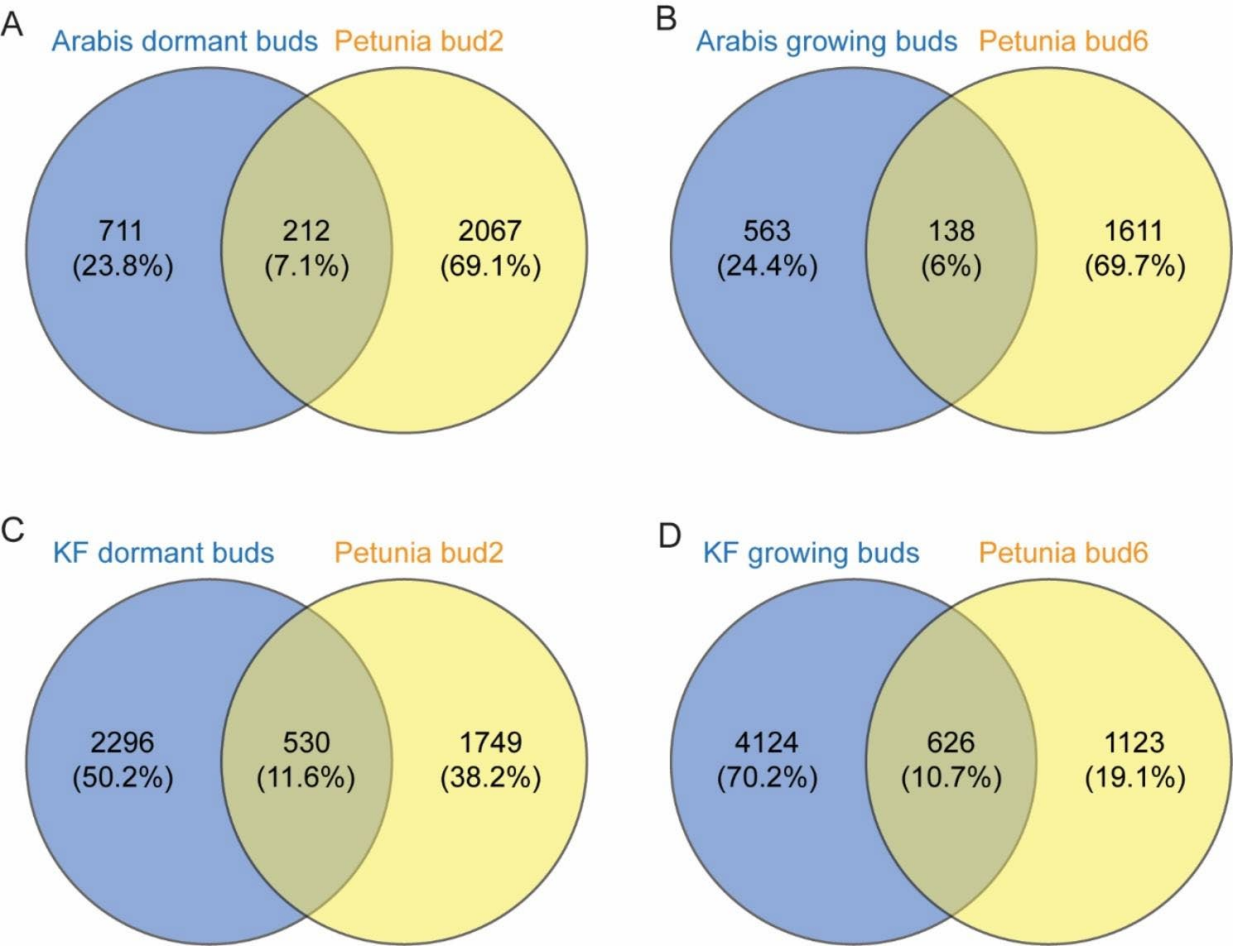
Identification of differentially expressed genes (DEGs) in common between buds from petunia, kiwifruit, and *Arabis alpina* axillary bud RNA-seq. **A**, comparison of the significantly upregulated genes in dormant buds from *Arabis alpina* (genes highest in V2 buds 5 d post-vernalization, 55) and petunia (genes highest in bud 2 from high phosphate samples in the first experiment). **B**, comparison of the significantly upregulated genes in growing buds from *Arabis alpina* (genes highest in V3 buds 5 d post-vernalization, 55) and petunia (genes highest in bud 6 from high phosphate samples from the first experiment). **C**, comparison of the significantly upregulated genes in dormant buds from kiwifruit (KF dormant, genes high in June harvested buds, 56) and petunia (genes high in bud 2 from high phosphate samples in the first experiment). **D**, comparison of the significantly upregulated genes in growing buds from kiwifruit (KF growing, genes high in December harvested buds, 56) and petunia (genes high in bud 6 from high phosphate samples from the first experiment). Petunia RNA-seq data used here are from the DEGs (>|2|-fold, padj < 0.05) between bud 2 and bud 6 under high P conditions from the first experiment. Arabis DEGs (>|2|-fold, padj < 0.05) were from the comparison between V2 and V3 buds 5 days post vernalization [55]. The kiwifruit DEGs (>|2|-fold, padj < 0.05) were generated from a contrast between the normalized counts from December and June using DESeq2 package [56]

In *Arabis alpina*, axillary buds at the V2 zone are dormant despite cold treatment, while axillary buds at the V3 zone grow out after vernalization [55]. The authors identified 1984 significant DEGs between V2 and V3 buds. When we compared the genes that were upregulated in bud 2 (compared with bud 6) under high P in experiment 1 with the genes that were upregulated in the dormant V2 buds (compared with V3 buds) 5 days post-vernalization [55], 212 genes were in common (Fig. 5A and Table S7). Many of these common genes are involved in ABA metabolism and signaling, for instance, *ABI1*, *ABF2*, *ABI FIVE BINDING PROTEIN3* (*AFP3*), and *NCED3*. There were also 138 genes in common between the active V3 buds from *A. alpina* and petunia bud 6: several of these common genes are cell cycle- and cell division-related genes, as well as an auxin efflux carrier, *PIN6* (Fig. 5B and Table S8).

A kiwifruit axillary bud time-course dataset has previously been described by Voogd and colleagues [56], in which the authors sampled the buds monthly and compared the transcriptome changes between dormancy onset, dormancy release, and budbreak. We generated a DEG list between the kiwifruit axillary buds from December (growing) and June (dormant) and compared the upregulated genes in dormant kiwifruit buds to the upregulated genes in petunia bud 2; and compared the upregulated genes in growing kiwifruit buds to the upregulated genes in petunia bud 6. There were 530 genes in common between dormant kiwifruit buds and petunia bud 2 (Fig. 5C) including SL, ABA, and dormancy-related genes, such as *DWARF14* (*D14*) (*DAD2* ortholog), *NCED3*, *NAP*, and *DRM1* (Table S9). There were 626 genes in common between growing kiwifruit buds and petunia bud 6 (Fig. 5D) including multiple cell-cycle and growth-related genes, such as *CYCLINs*, *SCARECROW-LIKE 28* (*SCL28*), *GROWTH-REGULATING FACTORs* (*GRFs*), and *PROLIFERATING CELL NUCLEAR ANTIGEN2* (*PCNA2*) (Table S10).

We also compared the upregulated genes in petunia bud 2, *A. alpina* V2 buds and dormant (June harvested) kiwifruit buds. There were 76 genes common to all three datasets, including ABA and dormancy-associated genes that were mentioned above (Table S11). When we compared the genes upregulated in the active axillary buds from these experiments, we found 82 genes in common to all three datasets, including cell-cycle and growth markers, such as *CYCs*, *CDKs*, and *PCNA2* (Table S12).

**Fig. 6:**
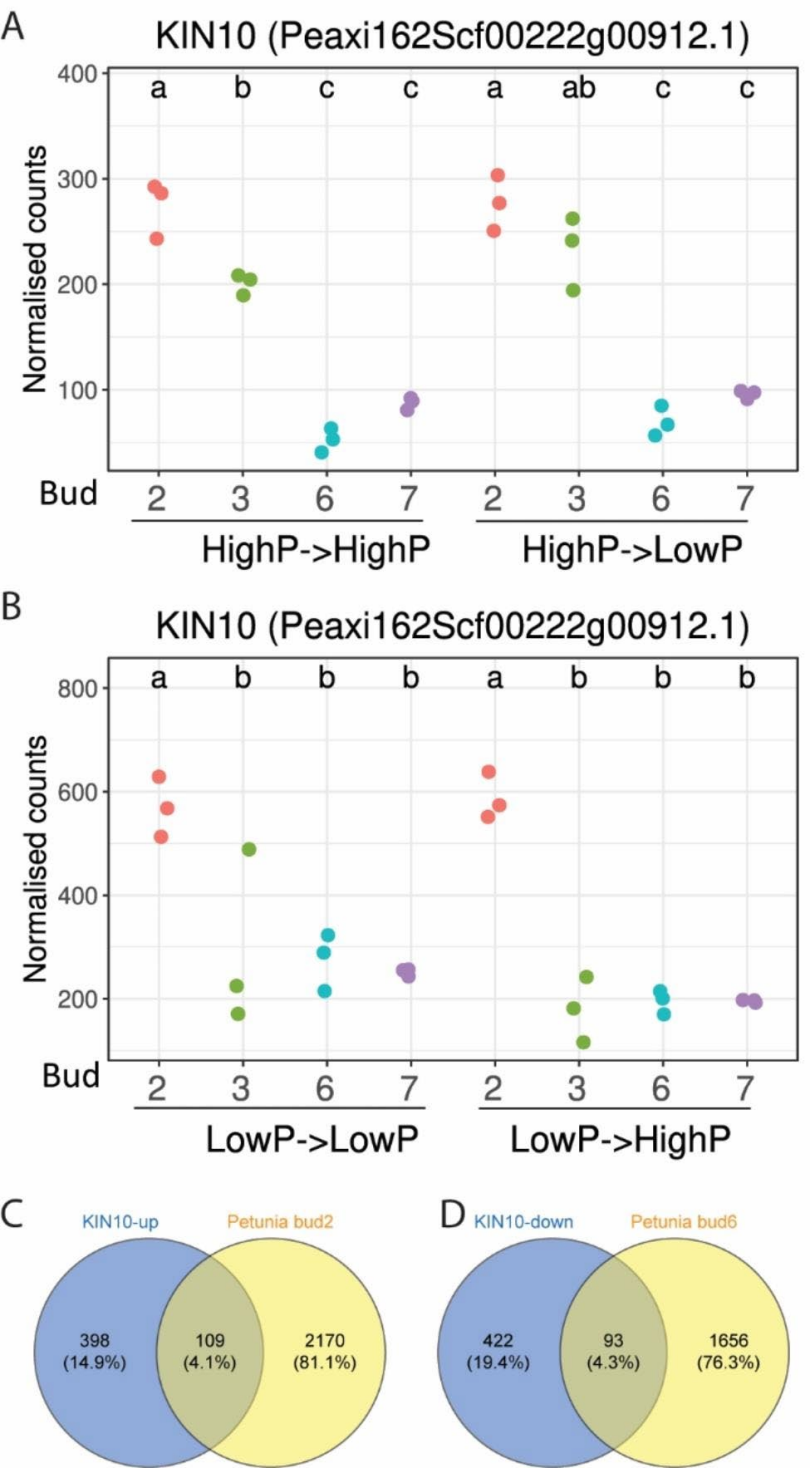
Identification of differentially expressed genes (DEGs) in common between AtKIN10 regulated genes [59] and genes upregulated in petunia bud 2 or bud 6. **A-B**, a petunia homolog of AtKIN10 was up-regulated in basal axillary buds from both experiments (A, high to low phosphate (P); and B, low to high P). Normalized counts and DEGs between bud 2 and bud 6 were generated from DESeq2 package and the different letters refer to the significance (*p* < 0.05) between samples calculated with Tukey honest significant differences method (Tukey HSD). **C**, common genes were found between genes upregulated after AtKIN10 induction in Arabidopsis protoplasts (>2-fold) and genes upregulated in petunia bud 2 compared with bud 6 from the first experiment (high phosphate samples). **D**, common genes were found between genes downregulated after AtKIN10 induction in Arabidopsis protoplasts (>2-fold) and genes upregulated in petunia bud 6 compared with bud 2 from the first experiment(high phosphate samples)

It has been suggested that sugar or carbon (C) availability contributes to axillary bud outgrowth, and axillary bud growth suppression might be a consequence of carbon deprivation/starvation [46, 47, 57, 58]. We used our RNA-seq data to explore the possible correlation between growth suppression in bud 2 and the carbon supply to these buds. In Arabidopsis, SnRK1/AtKIN10 is a central regulator of carbon deprivation responses, and its expression is suppressed by sugar [59–61]. Evidence suggested genes that were regulated by AtKIN10 largely overlapped with those that were altered by either carbon starvation conditions or sugar treatments [59]. We found two petunia genes encoded proteins with 80% homology to Arabidopsis AtKIN10, one of which had 2- to 5-fold more transcripts in bud 2 compared with bud 6 in experiments 1 and 3 (Fig. 6A and B). We compared the genes that were upregulated in bud 2 (> 2-fold compared with bud 6) under high P from the first experiment with the genes that were induced (> 2-fold) by expression of *AtKIN10* [59] and identified 109 genes in common (Fig. 6C and Table S13). These common genes included stress response, and hormone signaling and response genes (Table S13). On the other hand, there were 93 common genes between AtKIN10 downregulated genes from Arabidopsis and genes that were downregulated in bud 2 compared with bud 6 (or upregulated in bud 6 compared with bud 2) (Fig. 6D and Table S14).

### WGCNA identifies candidate genes that may be involved in regulating bud growth

**Fig. 7:**
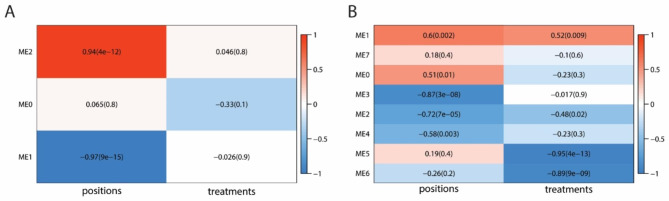
Co-expression analysis with Weighted Gene Co-expression Network Analysis (WGCNA). **A-B**, the module-traits (ME: module eigengenes; positions: buds 2, 3, 6, and 7; treatments: phosphate (P) treatments) relationship in the first experiment (high to low P, A) and in the third experiment (low to high P, B). The number in the box represents correlation between the module and the bud position or P treatment, and the number in the bracket represents the *p*-value for that correlation. Red indicates a strong correlation with higher bud position (e.g., bud 7) and high P. Blue indicates a strong correlation with lower bud position (e.g., bud 2) and low P

To further investigate genes that may be involved in regulating bud growth, we used Weighted Gene Co-expression Network Analysis (WGCNA) [62] to construct co-expression networks from gene modules. We filtered out the genes that had less than 50 mean counts and selected the top 25% of genes (~ 5000 genes; those where transcript levels varied the most between samples) for network construction. The analysis produced a small number of modules, 3 and 7 modules, respectively, for each experiment (Fig. 7A and B). The co-expression network produced modules correlated to bud position, but not P treatment in the first experiment (Fig. 7A), whereas for the third experiment, the network produced modules that correlated to P treatment as well as bud position (Fig. 7B). The modules that correlated with bud position were relatively large (~ 2000 to 3000 genes) making it difficult to visualize the connection between many genes. Thus, we further clustered the big modules into smaller sub-clusters (4 clusters for ME1 and 5 clusters for ME2 in the first experiment; and 8 clusters for ME1 and 6 cluster for ME2 in the third experiment) and were able to visualize these clusters with Cytoscape (https://cytoscape.org/).

For the first experiment, although module ME2 was correlated with bud 6 overall, key SL signaling genes *BRC1* and *DAD2* were grouped into one of the clusters (cluster 1), and genes in this cluster had higher expression in bud 2 compared with bud 6. Genes in this cluster include several dormancy-related genes (e.g., *DRM1*, *ABF2*, and *ABA4*)(Figure S7A), suggesting this is a cluster for dormancy genes. Within this cluster, we found transcription factors (TFs) that are potential candidates for future study, such as MYB59, WRKY33, NAC1, NAC3, and a T-box TF (AT1G26620). When we examined the DAD2 and PhTCP3/BRC1 subnetworks by only selecting the genes connected to these two genes, MYB59, NAC1 and the T-box TF were all present within the subnetworks (Figure S7B).

For the third experiment, ME5 and ME6 were correlated with P treatment, and the co-expression network of ME5 had several P starvation genes, e.g., *SPX1-3*, *PHOSPHATE TRANSPORTERs* (*PHTs*), and *PURPLE ACID PHOSPHATASEs* (*PAPs*), as well as several ABA related genes, including *MOTHER OF FT AND TFL1* (*MFT*), *SULFATE TRANSPORTER3;4* (*SULTR3;4*), and *REGULATORY COMPONENTS OF ABA RECEPTOR1* (*RCAR1*) (Figure S8A). Many of the genes in this network were also reported as co-regulated in the ATTED-II database and in other studies [63–65]. From the third experiment we could identify smaller clusters within the big modules and found two dormancy related clusters from ME2 (Figure S9A-B). Interestingly, the SL biosynthesis gene *CCD7* and transporter *PDR1* were found in ME4 (Figure S8B). Overall, the co-expression analyses were able to distinguish the differences between buds at different positions from both RNA-seq experiments and to identify the P response module when the plants were transferred from low P to high P conditions.

## Discussion

### Petunia basal and apical axillary buds possess different phenotypic and transcriptomic profiles

We used transcriptome data from axillary buds located at different positions to identify genes likely to be involved in controlling the potential for bud outgrowth. Our data showed that the basal axillary buds (especially bud 2) are very different to the apical axillary buds (e.g., buds 6 and 7) not only in their development (Fig. 1 and Figure S1) but also in their transcriptome profiles (Fig. 2 and Figure S3). We also showed that the transcriptome changes caused by growth promoting conditions (e.g., high P) on apical axillary buds can be detected 24 h after transferring the plants from low P to high P medium.

The number of DEGs between bud 2 and bud 6 was sizable and largely in line with comparisons between growing and non-growing buds from other species, such as grape and kiwifruit [56, 66]. Almost all the cell cycle- and growth-related genes that were differentially expressed between bud 2 and bud 6 or 7 were highly expressed in buds 6 and 7, including *PCNA2*, *GRFs* and their interacting factors (*GIFs*), and many cell cycle genes (*CDKs* and *CYCs*) in both RNA-seq experiments (Fig. 3 and Table S1). Furthermore, transcripts associated with auxin and CK synthesis, signaling or response were more abundant when compared with the basal axillary buds. Similar findings were observed in growing axillary buds in *A. alpina*, kiwifruit and grape [55, 56, 67]. Cao et al. [68] also found that IAA (auxin) and CK levels in axillary buds were elevated significantly 3 h after decapitation in pea. By contrast, in petunia, the basal axillary buds, especially bud 2, had more transcripts from genes associated with responses to stress and external challenges, and catalytic and transcription activities (Fig. 2G and Figure S3B). The transcripts of WRKY TF, NAC TF, HEAT STRESS TF and LEA family members were more abundant in these buds (compared with buds 6 and 7, Table S1), which was consistent with the findings in dormant buds of grape and kiwifruit (Fig. 5) [56, 66, 67]. These gene families are generally thought to play a role in stress tolerance and response [69–72]. The transcripts of SL synthesis, transport and signaling genes were generally more abundant in bud 2 compared with bud 6 or 7 in experiments 1 and 3 (Fig. 3), supporting their inhibitory role in branching. The transcripts of ABA related genes were upregulated strongly in bud 2 (Fig. 4), implying a higher level of ABA in these buds or a greater sensitivity to ABA. Studies from other species had similar results from axillary buds entering/during dormancy or that had dormancy induced using low R:FR treatments [10, 56, 66, 67].

When we compared the genes that were upregulated in petunia bud 2 to genes that were upregulated in dormant buds from *A. alpina* and kiwifruit, we found many genes in common, including a number of ABA, stress and dormancy related genes (Fig. 5, Tables S7, S9 and S11). Also, many of the homologs of Arabidopsis dormancy genes [54] were highly expressed in bud 2 relative to bud 6 (Table S6). Together, our data suggested a different regulatory network of hormone and metabolite synthesis and signaling in axillary buds at different positions, and which led to the petunia apical axillary buds that were in most cases actively growing whereas the growth of basal axillary buds were suppressed.

### The growth suppression of petunia bud 2 is associated with carbon starvation

It has been suggested that carbon limitation and preservation could be a reason why buds enter into or stay in dormancy [47] and sugars are known to be required for shoot branching [73]. Many reports have characterized carbon starvation genes, such as *DRM1*, *SENESCENCE1* (*SEN1*), *ASN1/DIN6*, *EXORDIUM-LIKE1* (*EXL1*) and *LYSINE-KETOGLUTARATE REDUCTASE* (*LKR*) [74–77]. In addition, a report found that inducing the expression of AtKIN10 resulted in gene expression profiles of Arabidopsis protoplasts appearing similar to those altered either by carbon starvation conditions or sugar treatments [59]. In petunia bud 2, several of these carbon starvation genes were upregulated (Figure S6 and Table S13). Furthermore, many genes were in common between the AtKIN10-induced genes and genes that were upregulated in bud 2, including genes involved in sugar metabolism, signaling and transport (Fig. 6 and Table S13).

Tarancón and colleagues [47] used a set of dormancy associated genes [54] to identify co-regulated genes, grouping them into four gene regulatory networks (GRNs) and finding they were enriched with genes corresponding to carbon starvation response. Forty-four petunia homologs of these dormancy genes were differentially and highly expressed in bud 2, with most of them belonging to GRNII (ABA related) and IV (sucrose starvation response) (Table S6). These analyses suggest the growth suppression of bud 2 was correlated with a limited supply of carbon (i.e. C starvation). Presumably this limitation is due to restricted supply into the buds, particularly as undeveloped buds tend to not have well established vasculature connecting to the rest of the plant.

It can be difficult to determine whether carbon starvation is the cause or one of the consequences of the inhibition of growth. In our experiments, petunia plants had sufficient light and nutrients, but some buds still did not grow, presumably as one mechanism to safeguard against possible loss of stem tissues. Decapitation above node 3 in WT petunia led to outgrowth of buds 1–3 and even axillary buds from the cotyledons (Figure S1A), suggesting it is less likely that the dormancy of bud 2 was caused by global carbon limitation, as the decapitated plants only had three small leaves to supply energy compared with the intact plants. It is expected that there are other factors that contribute to the dormancy of bud 2, including branching inhibitory hormones (SL and ABA), competition for resources from other organs [42, 78], and maximization of light capture [79]. The observation that buds 2 and 3 can grow out after decapitation (Figure S1A) and in SL mutants [7], indicates that these buds are dormant only under standard conditions. If this is the case, carbon starvation perhaps is a consequence of the growth suppression of these buds.

### ABA likely contributes to growth suppression of axillary buds, especially under limited nutrient supply

Petunia SL mutants have increased branching compared with WT plants; however, branching can be reduced when the plants are grown in nutrient deficient conditions (both phosphate and nitrogen) [25], suggesting there is additional control, apart from SL, for shoot branching. It has long been suggested that auxin and CK play an antagonistic role in branching; however, the role of ABA in branching has not been intensively investigated until more recently [10, 27, 52]. Under limited nutrient supply, SL production (at least in plant roots) is increased, which also likely contributes to a reduction in branching [25, 80, 81]. Reports on the connection between nutrient supply and ABA levels in plants were far and few between until recently. Zhang et al. [53] showed that P starvation increased the expression of ABA biosynthesis genes and ABA content in Arabidopsis seedlings.

This potential link between P starvation and ABA signaling in plants is consistent with our data. We found that transcript levels of many ABA-associated genes changed in response to P level, suggesting ABA might contribute to branching regulation during nutrient limitation (Fig. 4 and Figure S5). We did not see this response in the first experiment as it was unlikely the plants were undergoing P starvation at the time samples were taken for RNA analysis (Fig. 4A). However, in the third experiment, where the plants started in low P medium, the transcript abundances of many ABA-associated genes were reduced significantly after only 24 h of high P treatment compared with the expression from plants that remained in low P conditions.

There is evidence suggesting ABA acts downstream of SL in branching regulation in Arabidopsis and rice [27, 52]. González-Grandío et al. [27] reported that induced expression of *BRC1* increased the expression of *NCED3* and ABA levels in Arabidopsis seedlings and ABA application to rice SL mutants inhibited the growth of axillary buds [52]. However, in tomato ABA biosynthetic mutants, the expression of *CCD7* and *CCD8* was suppressed, and the SL levels were also reduced, suggesting a role for ABA in regulating SL biosynthesis [50].

Our data provide some support for the hypothesis that ABA modulates branching at least partially independently of SL/BRC1 signaling because many ABA-related gene transcripts had greater than 2-fold changes between P treatments in the third experiment (Figs. 3 and 4), but not a significant change in *BRC1* transcript levels. These results might explain why the SL mutants remain responsive to nutrient limitation [25]. In addition to SL and ABA, other hormones, especially CK, may contribute to this nutrient response [4, 23, 82, 83]. In our data, we found the transcript levels of *LOG8* and several CK response genes were significantly upregulated in buds 6 and 7 after 24 h of high P in the third experiment (Figure S4). Additionally, the transcripts of some of these genes were not significantly different between buds under the initial P limitation but became significantly different between bud 2 and bud 6/7 after 24 h of high P (Figure S4).

## Conclusions

Our work aimed to understand why some axillary buds are able or unable to grow even under favorable conditions and to identify genes that may be involved in promoting or inhibiting bud outgrowth. We showed that the phenotypic data correlated with the transcriptome differences between basal and apical axillary buds in petunia. Our data indicated that limited P supply increased the transcript abundance of ABA-associated genes in apical axillary buds, suggesting the branching suppression effect of low P might be mediated partially through ABA level in the buds. Higher transcript abundance of ABA- and dormancy-related genes within the basal axillary buds could explain the growth suppression of these buds and the growth suppression was correlated with a limited supply of carbon to these buds. Studying the branching pattern of mutants that are lacking SL and ABA signaling would provide evidence in understanding how SL and ABA coordinate the suppression of bud outgrowth. Candidates that were identified in this work will be the focus of future work to investigate their ability to alter shoot branching.

## Materials and methods

### Plant material and growth conditions for hydroponic experiments

All plants used in this work were the standard laboratory variety *Petunia hybrida* (inbred line V26) as previously described [25], with seed generated as needed in this laboratory. These plants were grown following protocols previously described [25]. In brief, seeds were germinated on seed raising mix (Dalton, Matamata, New Zealand) with a thin layer of vermiculite. When the seedlings were about 18 days old, around 90 similar size seedlings were transferred into individual baskets with clay balls and suspended in a 20 L container (four containers in total) containing hydroponic solution (nutrients as per [25] with either high phosphate, 250 µM; or low phosphate, 5 µM; depending on the experiment) with continuous aeration. The pH of the hydroponic solution was checked every two days and maintained at around 5.7.

The hydroponic experiments were carried out in a glasshouse unit at 22–24 °C with natural lighting (in Auckland New Zealand) and supplemented from LED lights (model LX601c, Heliospectra, Gothenburg, Sweden). The supplemental lighting was turned on from 5 to 10 pm and 6 to 9 am each day to maintain a relatively stable long day condition. The photon flux density was measured on two occasions. The first measurement was on an overcast morning around 8.30 am with the LED lighting: the photon flux densities of six positions on the bench where the plants were located was between 213 and 384 µmol m^− 2^ s^− 1^, with an average flux of 309 µmol m^− 2^ s^− 1^. The photon flux density for the second measurement was on a sunny afternoon (with non-direct sunlight): the densities for the same six positions ranged from 180 to 290 µmol m^− 2^ s^− 1^, with an average of 235 µmol m^− 2^ s^− 1^.

In total, three hydroponic experiments were performed from late June to early October 2020. All hydroponic solutions contained a range of nutrients with either high or low phosphate [25]. In the first two experiments, about 90 petunia seedlings (18 days old) were transferred into medium containing high phosphate (high P, 250 µM), and later were split into two groups once the plants had developed 9–10 true leaves (16–18 days in hydroponics). One group was transferred into fresh high P solution and the other group was transferred into low P (5 µM) solution. In the third experiment, a similar number of seedlings (18 days old) were transferred into low P solution and grew until they developed 9–10 true leaves (18 days in hydroponics). They were then split into two groups; one group was transferred into fresh low P solution and the other group was transferred into high P solution.

### Petunia branching phenotype from hydroponic experiments

Most of the plants from these experiments were used for tissue collection and subsequent RNA-seq. Seven to eight plants from each group were kept intact in their solution for phenotyping one week after the plants were transferred into fresh high P and low P solutions. Branch growth from each group was measured by counting the number of leaves (> 5 mm length) visible on the axillary bud/branch at each node along the main stem of the plant. In the first experiment, the number of leaves was recorded from buds at nodes 2, 3, 6–8. For the second and third experiments, the number of leaves was counted from nodes 1 to 10.

### Tissue collection, RNA extraction, and RNA-seq

For all three experiments, axillary buds from nodes 2, 3, 6 and 7 were excised using a scalpel at 3 and 24 h after transferring to fresh solutions and placed immediately into liquid nitrogen. Three biological replicates were collected, with each replicate consisting of a pool of axillary bud tissues from 6 to 7 plants. For each experiment, a total of 48 samples were collected: two time points (3 and 24 h), two treatments (high P and low P), three replicates, and four bud positions (bud 2, 3, 6 and 7).

Frozen tissues from the first (high-to-low P) and third (low-to-high P) experiments were ground into fine powder using a plastic pestle in a 1.5 mL tube. Total RNA was then extracted from the powder using the Spectrum™ Plant Total RNA kit (Sigma-Aldrich, St. Louis, MO, USA) according to the instruction manual and treated with DNase using On-Column DNase I Digestion Set (Sigma-Aldrich, St. Louis, MO, USA). The quantities and purity of the extracted RNA samples were measured using a NanoDrop™ 1000 Spectrophotometer (Thermofisher, Waltham, MA, USA) and RNA integrity was checked using the RNA 6000 Nano kit and a Bioanalyzer 2100 (Agilent Technologies, Santa Clara, CA, USA). RNA samples were dried within RNAstable tubes (Biomatrica, San Diego, CA, USA) or GenTegraRNA tubes (GenTegra, Pleasanton, CA, USA) for shipping to the sequencing provider.

All 48 RNA samples from the first experiment were shipped to AGRF (Australian Genome Research Facility, Melbourne, Australia) for library construction and 100 bp paired-end sequencing on a NovaSeq 6000 platform using a single lane of S4 flow cell (Illumina Inc., USA), which generated over 45 million reads depth per sample. For the third experiment, only 24 of the RNA samples (the 24 h time point samples) were sent for sequencing on the same platform with 150 bp paired-end on one lane of a S4 flow cell. This was due to the data from the first RNA-seq suggesting there were few transcriptomic differences between high and low P treatments 3 h after medium switching. The reads depth for this sequencing were close to or above 100 million per sample.

### Petunia decapitation experiment

Petunia WT seeds were germinated and grown on seed raising mix (Dalton, Matamata, New Zealand) with a thin layer of vermiculite. The seedlings were transferred into individual pots with potting mix two weeks after sowing and watered every 3–4 days with tap water. The decapitation experiment was carried out four weeks after sowing. Plants in these two groups (decapitation and intact) were at a similar developmental stage, with on average 7.1 and 6.8 leaves (n = 8 and 10) on the main stem, respectively (*p* = 0.48). For plants receiving decapitation treatment, the stem above node 3 (from the base) was removed. Branch growth (as number of leaves > 5 mm on each bud) of both groups was measured on day 5, day 8 and day 12 after decapitation.

### RNA-seq data processing

Raw RNA-seq data were processed using a Nextflow pipeline, nf-core (https://nf-co.re/rnaseq, version 1.4.2 for experiment 1 and version 3.0 for experiment 3). It used Trim Galore (version 0.6.6) (https://github.com/FelixKrueger/TrimGalore) to trim low-quality bases and adapters (Quality cutoff: 20; and Minimum sequence length: 20 bp). The pipeline subsequently checked the quality of raw data using FastQC, and performed genome alignment and quantified data using Salmon, with automatic library type detection [84]. The results from the QC of each sample were collated using MultiQC (version 1.9) [85] to allow easy comparison of QC metrics between all samples. The reads were aligned to the *Petunia axillaris* genome [86]. The normalized counts and DEGs between various contrasts were produced by DESeq2 [87], using the ashr shrinkage method [88]. Significant DEGs used a cut-off of 2-fold changes and adjusted *p*-value (padj) < 0.05. PCA plots and expression heatmaps were also generated from DESeq2 package after log transformation.

### Venn diagrams and GO enrichment analysis

The Venn diagrams were generated from Venny(https://bioinfogp.cnb.csic.es/tools/venny/*).* The petunia RNA-seq data used here are from the DEGs between bud 2 and bud 6 (high P samples) from the first experiment. *Arabis alpina* DEGs were obtained from the comparison between V2 (dormant) and V3 (growing) buds 5 days post vernalization [55]. The kiwifruit DEGs (>|2|-fold, padj < 0.05) were generated from a contrast between growing axillary buds in December (summer) and dormant buds in June (winter) using DESeq2 package. Arabidopsis data are derived from lists of genes that were either upregulated or downregulated by expression of *AtKIN10* in protoplasts [59].

GO enrichment analysis was carried out with TBtools [89]. Analysis settings included: multi-test adjustment method, BH (FDR); significant level, padj < 0.05; and gene ontology type, plant GO slim. Genes that are significantly differentially expressed (>|2|-fold, adjusted *p*-value < 0.05) between bud 2 and bud 6 were used for GO analysis.

### Gene expression by digital droplet PCR

First-strand complementary DNA (cDNA) samples were synthesized in 20 µL reactions using 0.5 µg of RNA with iScript Reverse Transcription Supermix (Bio-Rad, Hercules, CA, USA) following the manufacturer’s instructions. The cDNA samples were then diluted to 250 µL with water and the relative gene expression performed using a QX200 Droplet Digital PCR system (ddPCR, Bio-Rad, Hercules, CA, USA). Each reaction consisted of 4 µL of the diluted cDNA, 11 µL of EvaGreen supermix (Bio-Rad, Hercules, CA, USA), 100 nM of gene specific primers and water (total 22 µL) and was set up in a 98-well plate. The PCR conditions were as follows: initial denaturation at 95°C for 5 min, 40 cycles of denaturation at 96°C for 30 s, annealing and extension at 58°C for 45 sec, and signal stabilization at 4°C for 5 min and at 90°C for 5 min. No template controls (NTCs) for each target gene and two reference gene controls (GAPDH and ACTIN2) were assembled on the same plate for each run. Primers used for ddPCR include PhPT1 (5’-GGCAACTAATGACATGTCCA-3’ and 5’-GAACAAACCGAAGTCATTGC-3’), CDKB1 (5’-TTAGGAACCCCAACTGAGCA-3’ and 5’-GGAACATGAGAGGCCAAGTT-3’), DAD2 (5’- TAGGTGGGAAGAACACAGTGC-3’ and 5’-CCTATGTGAAAGAGCTCTTCTCAACTC-3’), TCP3 (5’- TGCAGTCAAGGAGCTGGAAG-3’ and 5’-TATCATTTGTGGCAGATTCGTC-3’), SPX2 (5’-GGAAGTTCAACTGTTAGCGA-3’ and 5’-TCGACCACTGGACTATTCTT-3’), SPX3 (5’- GAGAACAGGTGGATTACTGC-3’ and 5’-ATGGTGCTTTCACACTCTTT-3’), GAPDH (5’- GACTGGAGAGGTGGAAGAGC-3’ and 5’-CCGTTAAGAGCTGGGAGAAC-3’), and ACTIN2 (5’- CCTGATGAAGATCCTCACCGA-3’ and 5’-CAAGAGCCACATAGGCAAGCT-3’).

### Co-expression WGCNA analysis

Normalized counts from the 24 h time-point samples were used for WGCNA co-expression analysis [62]. The genes were filtered by a mean count of > 50 with only the top 25% most varied genes being used for network construction. Parameters for the network construction include: power = 12 (for the first experiment) or 16 (for the third experiment), maxBlockSize = 10,000, networkType = “signed”, TOMType = “signed”, minModuleSize = 30, corType = “pearson”, and mergeCutHeight = 0.1. Two big modules (~ 2000–3000 genes) from each experiment were clustered into smaller clusters using hierarchical clustering (method: complete) in R. The modules and clusters were exported to Cytoscape (https://cytoscape.org/, version 3.9.1) for visualization.

### Statistical analysis

Unless otherwise specified, statistical analysis was performed in R (version 3.6.0–4.0.0). A generalized linear model (GLM) was fitted with poisson distribution using the branch growth phenotype data and the statistical significance of the P treatment and P effect on each bud was calculated using Analysis of Variance (ANOVA) and post-hoc Tukey’s honestly significant difference (HSD) test, which corrects the Type I error rates from multiple comparisons. For transcript levels from RNA-seq, GLM was used unless the expression data were skewed, in which case the data were log2 transformed and a linear model was fitted. The statistical significance between samples was calculated using ANOVA and post-hoc Tukey’s HSD tests.

### Electronic supplementary material

Below is the link to the electronic supplementary material.

Supplementary Material 1

Supplementary Material 2

Supplementary Material 3

Supplementary Material 4

Supplementary Material 5

Supplementary Material 6

Supplementary Material 7

## Data Availability

The datasets generated and/or analyzed during the current study are available either in supplemental data files or in the Gene Expression Omnibus (https://www.ncbi.nlm.nih.gov/geo/) data repository (accession number GSE237985).
