# Supplementary material for: Transcriptomic analysis implicates ABA signaling and carbon supply in the differential outgrowth of petunia axillary buds
Source: BMC Plant Biol. 2023 Oct 10;23:482. doi: 10.1186/s12870-023-04505-3 (PMC10563266; doi:10.1186/s12870-023-04505-3)
Supplement: Supplementary file 7 — Supplementary Material 7 [file 12870_2023_4505_MOESM7_ESM.pdf]

Supplementary Figures 1-9

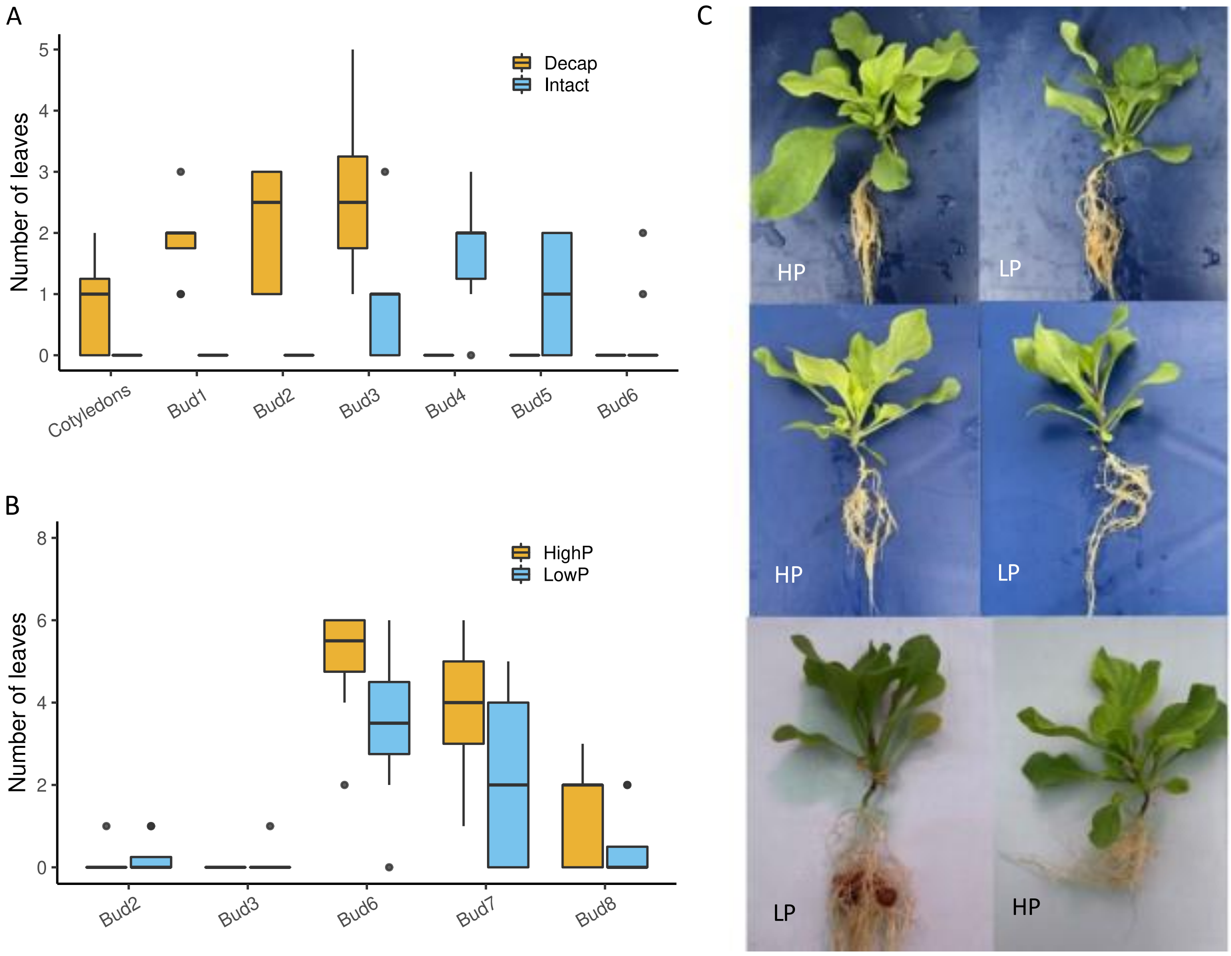

Figure S1. The branch growth of buds at different positions on the main stem were very different, and the growth was affected by P conditions.

A, the branch growth of intact (Intact) and decapitated (Decap) plants 33 days after germination and 5 days after decapitation. Plants between these two groups were at a similar developmental stage (similar number of leaves on the main stem) before decapitation and for the decapitation treatment, the stem above node 3 was removed. Error bars indicate SEM, n = 8-10.

B, branch growth (measured as number of leaves > 0.5 cm in length of buds 2 and 3, and buds 6-8) from the first experiment, where plants were grown in high P for 2-3 weeks then split into two groups. One group went into fresh high P, while the other group was transferred to low P media. The branching phenotype (numbers of leaf on each node) was measured at 7 days after the change of media.

C, representatives of plants at the time of phenotyping, 7 days after transferring into new media. The top and middle panels were from the first and second experiments (experimental conditions stated as above, HP: a plant that was transferred to high P, LP: a plant was transferred to low P), and the bottom panel was from the third experiment (conditions stated in Figure 1, LP: a plant that was transferred to low P, and HP: a plant was transferred to high P).

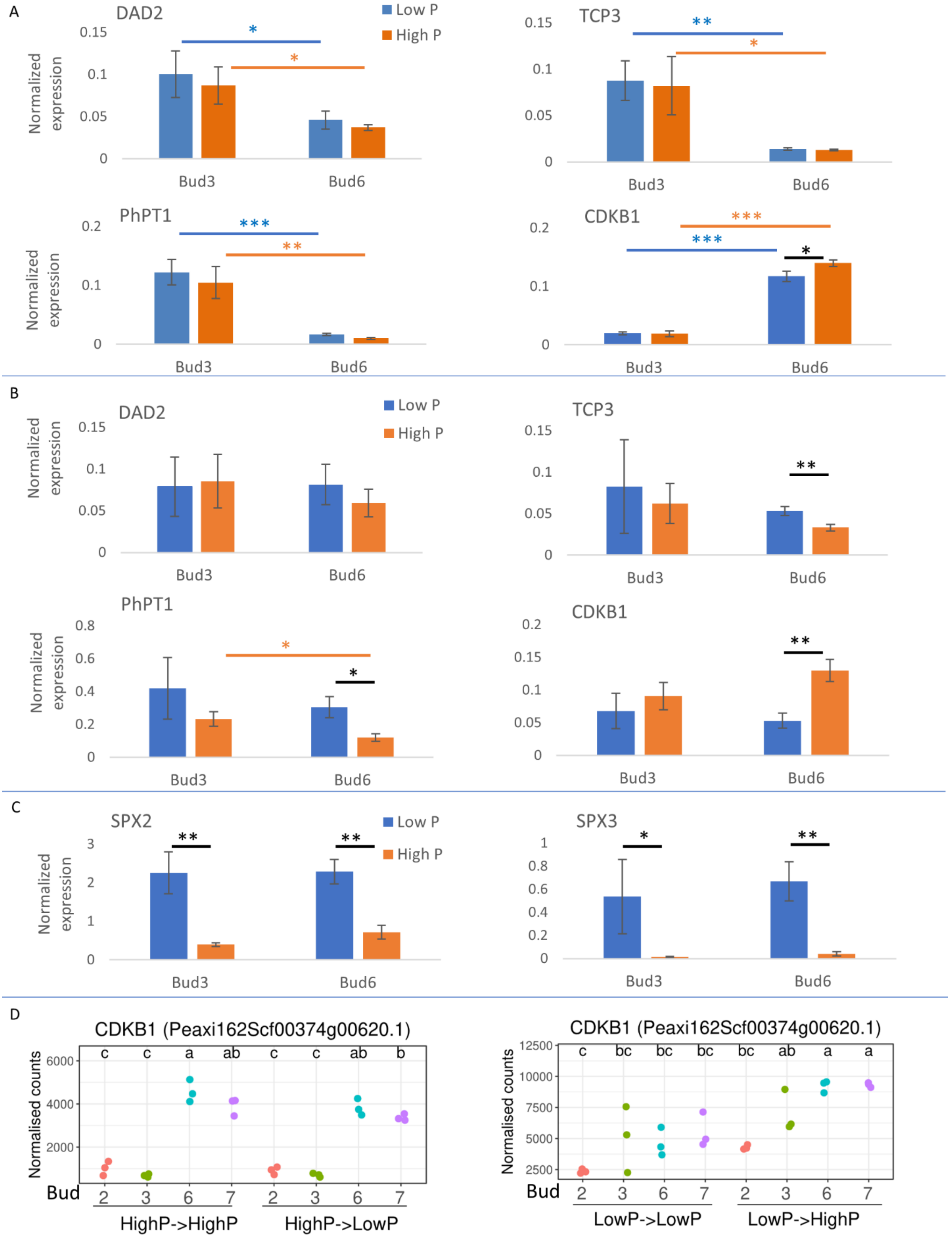

Figure S2. Transcript levels of a selection of genes from ddPCR and RNA-seq.

A – C, ddPCR of the 24 h samples of bud 3 and bud 6 from the first experiment (A) and from the third experiment (B and C). The values were normalized to the geometric mean of two reference genes, *ACTIN* and *GAPDH*, and the error bars are standard deviation (n = 3). The statistical significance between bud 3 and bud 6 and between P treatments on each bud were calculated using t-tests and the level of significance is indicated as follows: n.s, not significant; *, p < 0.05; **, p < 0.01; and ***, p < 0.001. D, transcript levels of *CDKB1* from the first experiment (left) and from the third experiment (right). The normalized counts were obtained from the R package DESeq2. The different letters refer to the significance ($p < 0.05$) between samples calculated with Tukey Honest Significant Differences method (Tukey HSD).

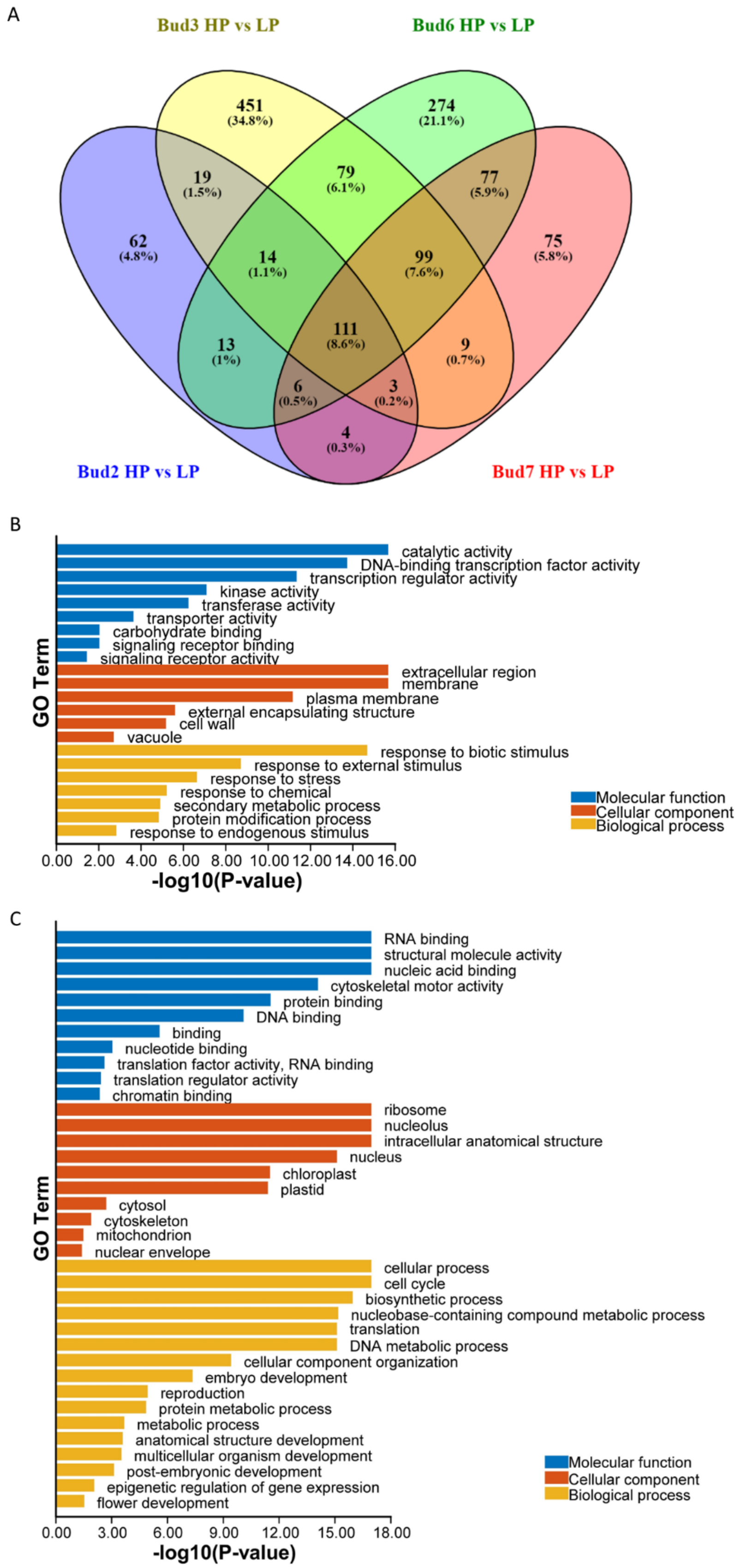

Figure S3. Venn diagram, GO enrichment, and KEGG pathway analyses.

A, Venn diagram of DEGs from the third experiment between high P and low P at 24h time-point on each bud position. The DEGs were generated with R package DESeq2 and the Venn diagrams were generated from Venny (*https://bioinfogp.cnb.csic.es/tools/venny/*).

B and C, GO enrichment analysis on genes that were highly expressed (fold changes > |2|, padj < 0.05) in bud 2 (B) and bud 6 (C) at 24 h time-point of low P condition (starting condition) from the third experiment. GO type, plant GO slim; statistical test method, Fisher; multi-test adjustment method: Yekutieli (FDR under dependency); significance level, 0.01.

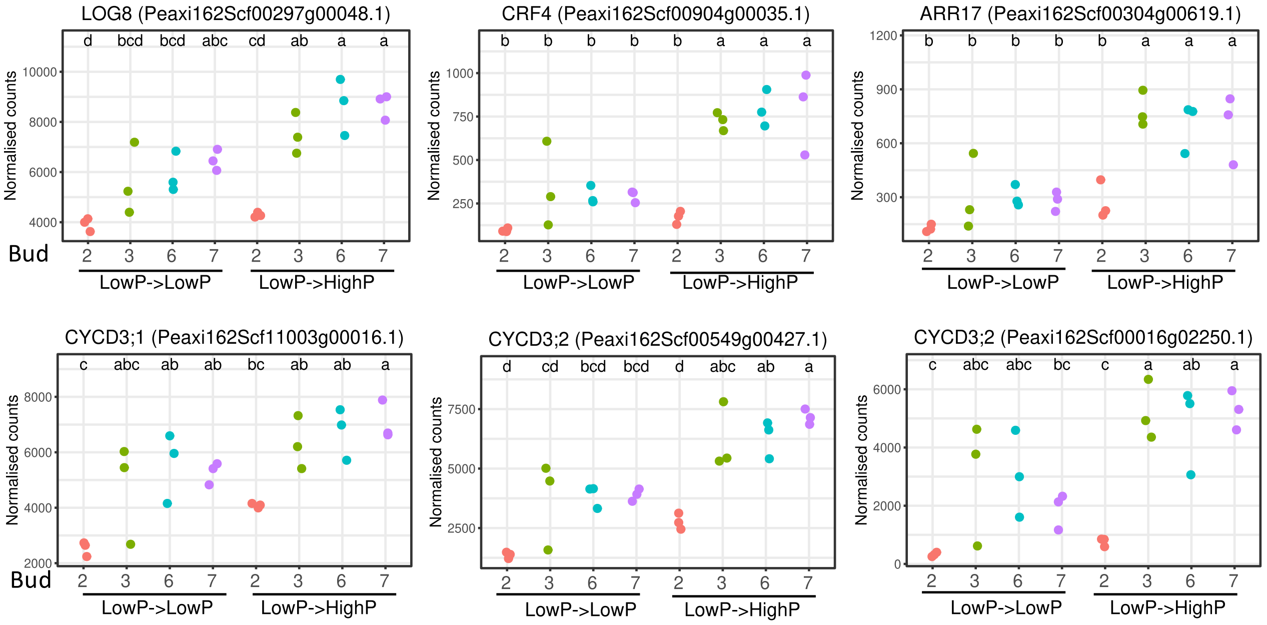

Figure S4. Expression of CK and cell cycle related genes that responded to high P at the 24 h time-point in the apical buds from the third experiment.

There are two *CYTOKININ RESPONSE FACTOR4* (*CRF4*) and two *RESPONSE REGULATOR17* (*ARR17*) homologs (only one is shown here) and all showed a similar pattern, in which the expression did not differentiate in low P between bud position but became significantly differentiated (>2-fold changes) between bud 2 and other buds after switching to high P for 24 h. The expression of *LONELY GUY8* (*LOG8*) *and CYCLIN D3;2* (*CYCD3;2*) were not different by more than 2-fold between buds and treatment, but the expression between high and low P on bud 6 were significantly different. The expression of *CYCD3;1* showed an increasing trend for all buds in the high P compared to the low P conditions, however, the differences were not significant. The normalized counts were obtained from the R package DESeq2. The different letters refer to the significance ($p < 0.05$) between samples calculated with Tukey Honest Significant Differences method (Tukey HSD).

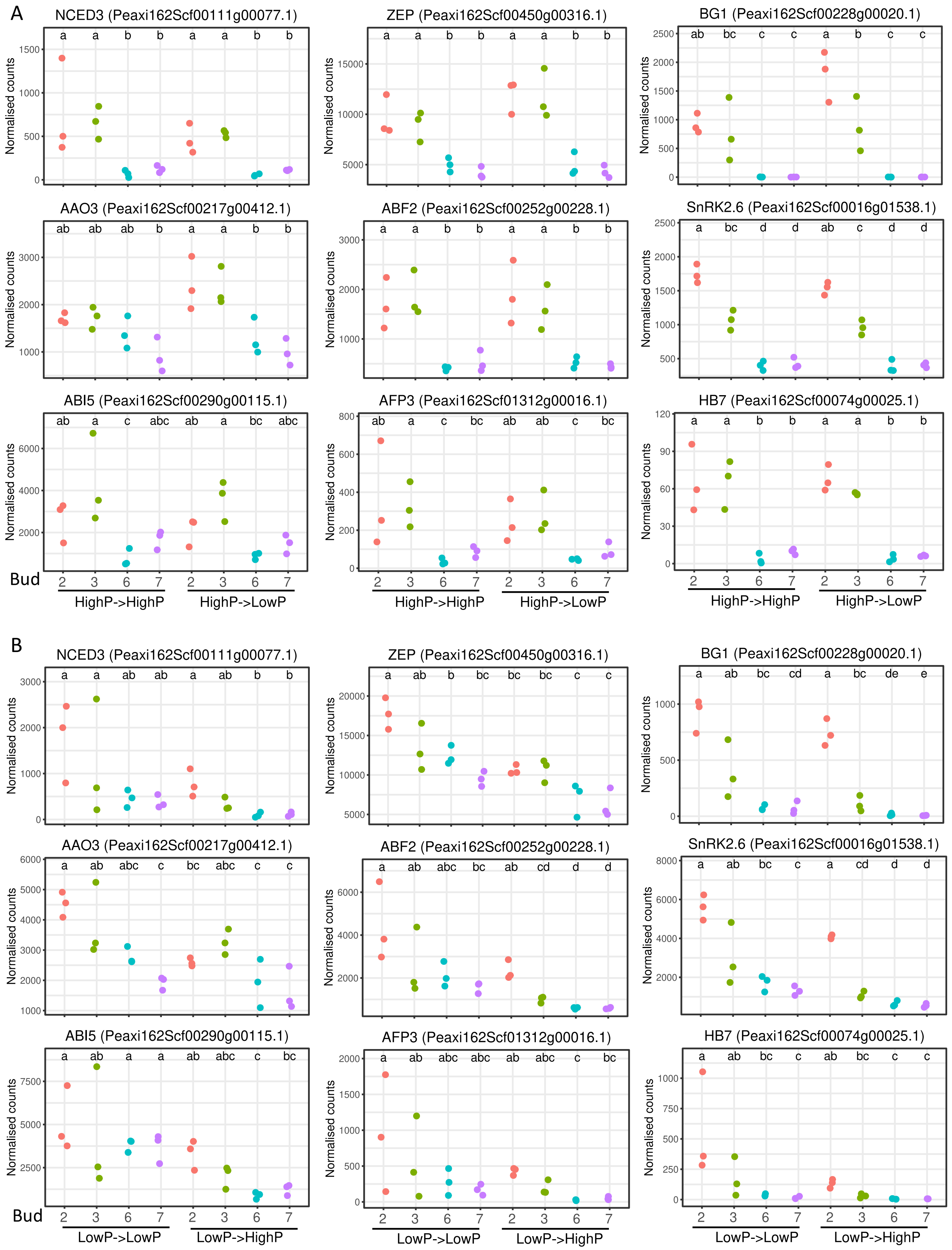

Figure S5. ABA synthesis, signaling, and response gene expression in petunia axillary buds.

A, expression pattern from the first experiment, and B, expression from the third experiment. Selected genes include *NINE-CIS-EPOXYCAROTENOID DIOXYGENASE3* (*NCED3*), *ABSCISIC ALDEHYDE OXIDASE3* (*AAO3*), *ABA INSENSITIVE5* (*ABI5*), *ZEAXANTHIN EPOXIDASE* (*ZEP*), *ABSCISIC ACID RESPONSIVE ELEMENTS-BINDING PROTEIN2* (*ABF2*), *ABI FIVE BINDING PROTEIN3* (*AFP3*), *BETA-GLUCOSIDASE1* (*BG1*), *SNF1-RELATED PROTEIN KINASE2.6* (*SnRK2*.6), and *HOMEOBOX7* (*HB7*). The normalized counts were obtained from the R package DESeq2. The different letters refer to the significance (p < 0.05) between samples calculated with Tukey HSD.

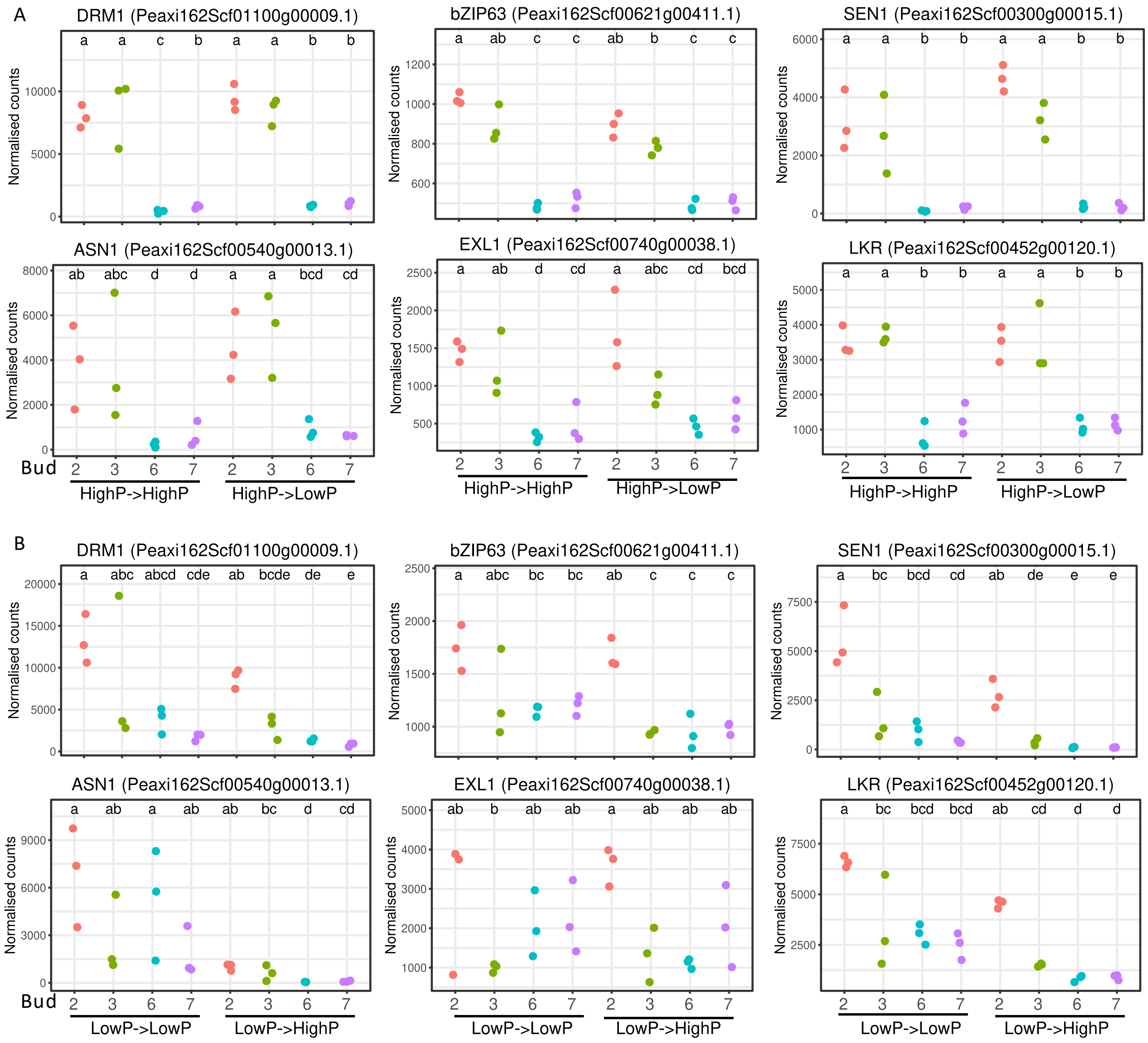

Figure S6. Expression pattens of some dormancy and C starvation related genes from the first experiment (A) and the third experiment (B).

Selected gene include *DORMANCY-ASSOCIATED PROTEIN1* (*DRM1*), *BASIC LEUCINE ZIPPER63* (*bZIP63*), *SENESCENCE1* (*SEN1*), *GLUTAMINE-DEPENDENT ASPARAGINE SYNTHASE1* (*ASN1*), *EXORDIUM LIKE1* (*EXL1*), and *LYSINE-KETOGLUTARATE REDUCTASE* (*LKR*).

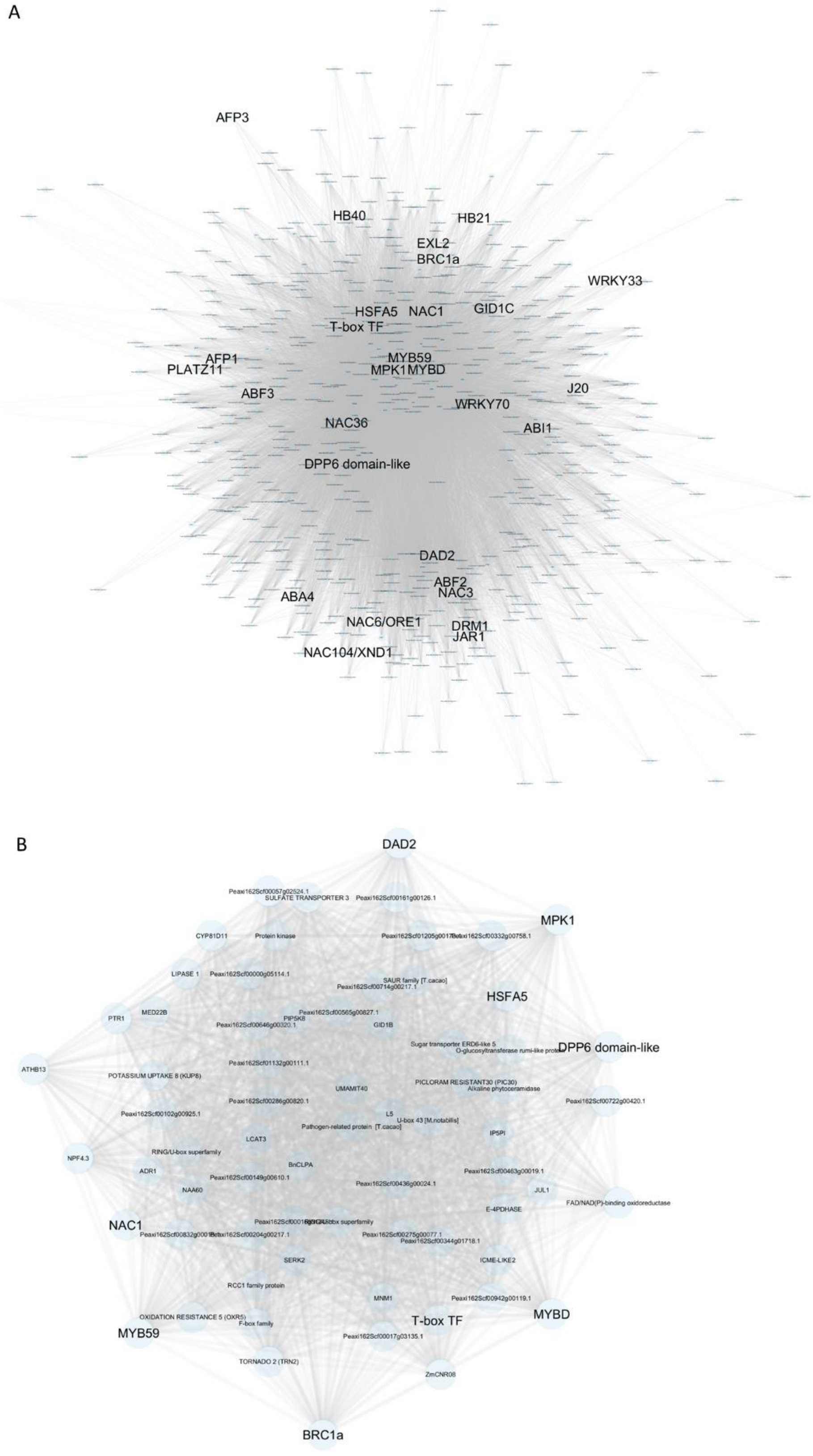

Figure S7. Co-expression analysis with WGCNA for the first experiment between bud 2 and bud 6

A, a network of dormancy genes (cluster 1) from module 2. Some known dormancy and ABA-related genes, as well as some TF genes were highlighted with larger text.

B, a sub-network of genes connected to DAD2 and BRC1 from the dormancy cluster in A.

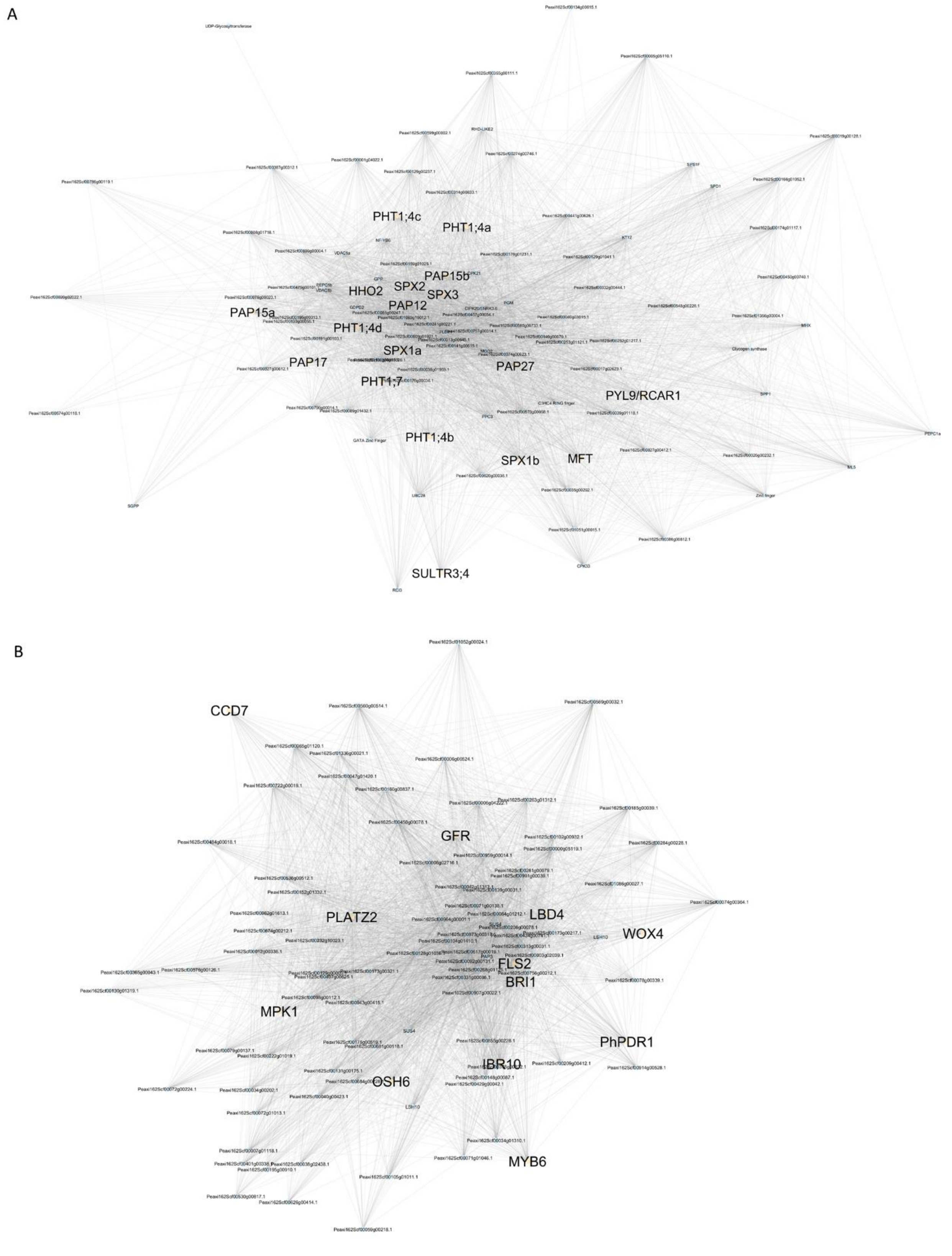

Figure S8. Co-expression analysis with WGCNA for the third experiment between bud 2 and bud 6

A, a phosphate related network from module 5 (ME5) in B (low to high P). The known P response and ABA-related genes were highlighted with larger text.

B, a module (ME4) that contains CCD7 and PhPRD1. Several transcription factors were highlighted with a larger text.

A

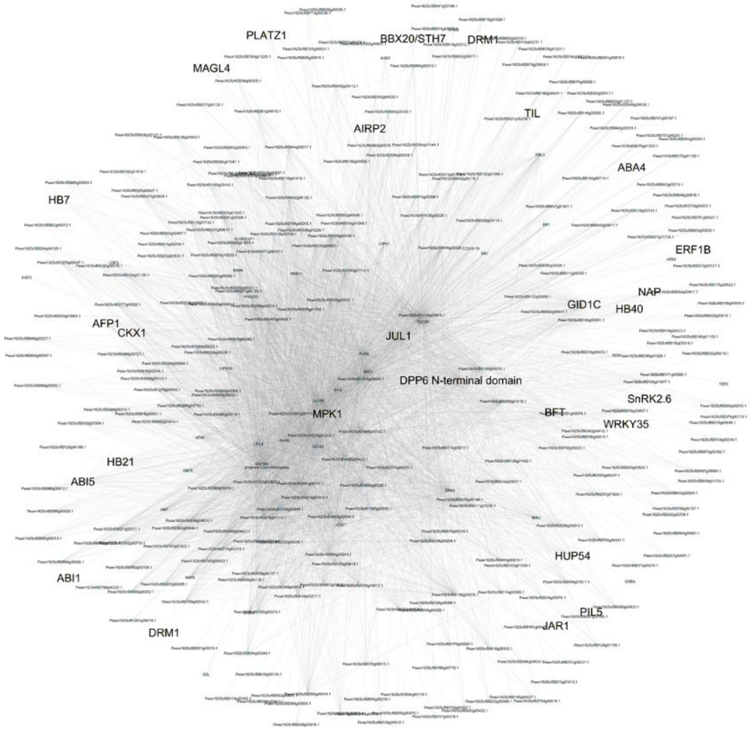

B

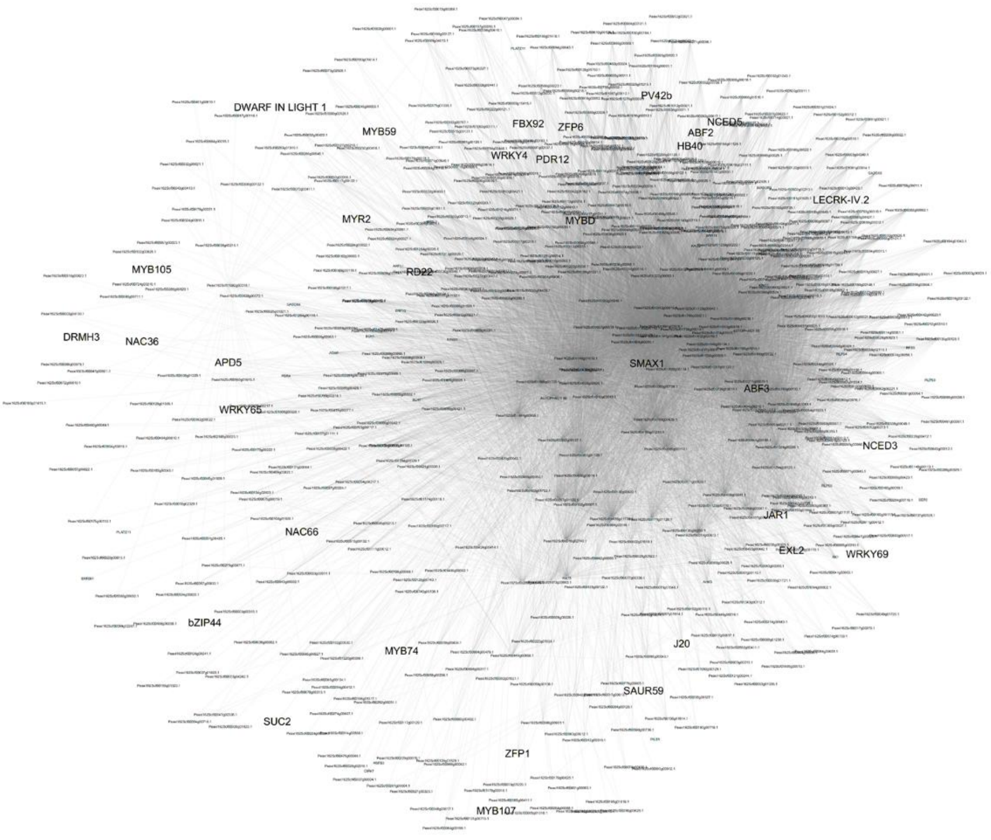

Figure S9. Co-expression analysis with WGCNA for the third experiment between bud 2 and bud 6

A and B, two dormancy clusters from the module ME2. Some known dormancy and ABA-related genes, as well as some TF genes were highlighted with larger text.
